# Identification of computationally designed skeletal and cardiac promoters with high specificity across AAV delivery routes

**DOI:** 10.1186/s12896-026-01141-1

**Published:** 2026-03-26

**Authors:** Nicole Zielinska, Erin L. Howard, Brenna A. Y. Stevens, Melanie M. Goens, Cici Yang, Elena S. B. Campbell, Madison E. Hughes, Yanlong Pei, Dinghai Zheng, D. Benjamin Gordon, Alec A. K. Nielsen, Raja R. Srinivas, Jeff L. Caswell, Luis G. Arroyo, Sarah K. Wootton

**Affiliations:** 1 Department of Pathobiology, University of Guelph, Guelph, Ontario Canada; 2 Department of Clinical Studies, Ontario Veterinary College, Guelph, Ontario Canada; 3 Asimov, Boston, MA USA

**Keywords:** AAV, Tissue-specific, Promoters, Algorithm, Targeted, Cardiac muscle, Skeletal muscle

## Abstract

**Background:**

Precise control of transgene expression is essential for safe and effective AAV gene therapies. While capsid engineering has advanced tissue targeting, progress in developing regulatory elements that confine expression to specific tissues has lagged. This gap limits the ability to minimize off-target expression and associated safety risks, emphasizing the need for improved tissue-specific regulatory control.

**Results:**

Here, we evaluated 12 computationally designed tissue-specific promoters generated by Asimov’s proprietary algorithm. Promoters driving a heat-stable human placental alkaline phosphatase reporter gene were packaged into AAV6·2FF and administered to C57BL/6 mice intranasally, intramuscularly, and intraperitoneally. Expression was assessed macroscopically, microscopically, enzymatically and by vector genome quantification. We identified two strictly skeletal muscle-specific promoters independent of the AAV capsid or route of administration used, and two cardiac-specific promoters when administered intraperitoneally.

**Conclusions:**

These findings demonstrate that computational promoter design can yield elements with great strength and precision that enable safer and more targeted AAV gene therapies and offer a broadly applicable strategy for tissue-specific expression in treating various monogenic diseases.

**Supplementary Information:**

The online version contains supplementary material available at 10.1186/s12896-026-01141-1.

## Introduction

With the growing number of AAV gene therapies entering clinical trials, safety and precision have become important considerations for AAV platform design [1]. Promoters are cis-acting regulatory elements within recombinant AAV genomes that govern the magnitude and cell-type specificity of transgene expression [2–5]. Accordingly, promoter selection is a key determinant of the transgene expression profile and should align with the intended target cell population. Yet, many of the current therapies rely on broadly active promoters, such as cytomegalovirus (CMV), chicken β-actin (CBA), or a chimeric chicken-β-actin promoter (CAG) made up from the cytomegalovirus immediate early (CMV) promoter, chimeric chicken-β-actin (CBA), and ubiquitin C (UBC) enhancer region to drive transgene expression [6, 7]. While these promoters have enabled the clinical translation of several therapies including the CMV promoter in Glybera for lipoprotein lipase deficiency [8], and the CBA promoter in Zolgensma for spinal muscular atrophy [9], their broad activity has been linked to several limitations. Some ubiquitous promoters are vulnerable to epigenetic silencing through CpG methylation, especially those of viral origin, which can compromise long-term transgene expression and reduce therapeutic efficacy [10–12]. Additionally, when a transgene expresses outside its intended tissue, it can trigger an immune response and increase the risk of adverse events [13–15]. In some cases, overexpression of a transgene can lead to toxicity, particularly when the vector is delivered systemically or when high doses of AAV are required to achieve therapeutic benefits [16, 17]. These limitations have sparked a growing interest in tissue-specific promoters as a means to achieve more precise and controlled gene expression.

Tissue-specific promoters are a promising alternative to ubiquitous promoters, by limiting transgene expression to a specific cell type or organ. They contain DNA sequences that bind to transcription factors (TFs) that are predominantly expressed in the tissue of interest [18, 19]. Their activity is also influenced by chromatin accessibility, DNA methylation and different enhancer interactions [20, 21]. These promoters can be derived from endogenous genes or can be synthetically engineered to include sequences and regulatory motifs that enhance tissue specificity and transcriptional control [22, 23]. However, many natural promoters lack the transgene expression levels needed for clinical efficacy, while synthetic promoters often require a lengthy process of trial and refinement to perform effectively [23]. Identifying the independent elements responsible for tissue specificity has traditionally relied on low-throughput and labour-intensive methods, making it difficult to design synthetic promoters quickly and reliably [23]. In response, computational and algorithmic approaches have emerged as powerful tools to provide a faster way to design tissue-specific promoters.

Through the recent advancements in synthetic biology and machine learning, researchers can now analyze large-scale genomic and transcriptional data sets to identify regulatory motifs and transcription factor binding sites that govern tissue-restricted gene expression [24–31]. To test the potential of these innovations, we collaborated with the company Asimov, which has developed a proprietary algorithm known as AAV-Edge. This algorithm combines multi-omics analysis and machine learning to identify and engineer promoter sequences optimized for expression in specific tissues in the body, while minimizing expression in off-target organs [32]. Using AAV-Edge, Asimov generated a series of 12 computationally derived promoters designed to drive cardiac and skeletal muscle-specific expression.

To validate these novel promoter designs, we packaged each construct into AAV6.2FF, a next-generation capsid with strong tropism for lung and muscle when delivered intranasally or intramuscularly, respectively, yet capable of transducing multiple organs following systemic administration [33]. Each promoter drove the expression of the human placental alkaline phosphatase (AP) reporter gene, which allowed for the visualization and quantification of AP expression across different tissues. Mice received the vector via intranasal (IN), intramuscular (IM), and intraperitoneal (IP) administrations to ensure that every major tissue had an opportunity for direct and indirect vector exposure, allowing us to evaluate whether promoter activity remained tissue-specific independent of the route of administration used. Promoter activity was evaluated both macroscopically and microscopically following AP staining of tissues and quantitatively via an enzymatic AP activity assay. Promoters demonstrating tissue-specific activity were subsequently tested with AAV9, a serotype characterized by broad tissue tropism and efficient systemic transduction [34, 35]. Promoters that showed strong transgene expression in their intended target tissue but also showed expression in a limited number of off-target tissues when delivered via all three routes were subsequently re-evaluated using only the IP route.

## Methods

### AAV plasmid generation

Twelve putative tissue-specific promoters were identified using Asimov’s proprietary algorithm. This algorithm used multi-omics and an AI-powered design to identify regulatory elements with activity in either the cardiac muscle, the skeletal muscle or in both cardiac and skeletal muscle. To evaluate these promoters, the twelve AAV genome plasmids were constructed, each encoding the human placental alkaline phosphatase (AP) reporter gene under the control of these computationally derived promoters. Although promoter sequences cannot be disclosed for intellectual property reasons, the computationally designed promoters evaluated in this study ranged approximately from 274 to 1517 bp in length. When combined with the WPRE, SV40 polyadenylation signal, and flanking AAV2 ITRs, these promoter sizes remain well within the AAV packaging capacity, allowing for transgene inserts of approximately 2.0–3.3 kb within the standard ~4.7 kb packaging limit. Each construct contained the woodchuck hepatitis virus post-transcriptional regulatory element (WPRE) and simian virus 40 polyadenylation (SV40) sequence bound by two AAV2 inverted terminal repeats (ITRs) at each end. For our positive control we generated an additional plasmid using the broadly active CASI promoter that contains the cytomegalovirus (CMV) enhancer, the chimeric chicken-β-actin (CAG) promoter and a ubiquitin C (UBC) enhancer region. All plasmid constructs were verified through full plasmid sequencing using Plasmidsaurus.

### AAV vector production and titration

AAV6.2FF vectors were produced using a dual-plasmid transfection approach in adherent HEK293 cells and purified via heparin affinity chromatography following a previously established procedure [36]. Alternatively, AAV9 vectors were produced using a triple plasmid transfection approach in adherent HEK 293 cells. For a single preparation of AAV9, 40 culture dishes (15 cm) were transfected with a total of 44 µg of DNA, including 11 µg of transgene plasmid, 11 µg of pAAV9 plasmid, and 22 µg of pAdDeltaF6. Polyethyleneimine (PEI Max) was used at a 3:1 PEI: DNA ratio to facilitate the delivery of the genetic material into the cells. At 4 days post-transfection, the cells and supernatant were harvested and centrifuged at 20,000 ×g for 30 minutes (min) at 4 °C. The supernatant was discarded and 15 mL of lysis buffer (150 mM NaCl, 50 mM Tris-HCl pH 8.5, 2 mM MgCl₂) was added to the cell pellet and lysed via three freeze-thaw cycles. The suspension was then centrifuged at 2000 ×g for 15 min at 4 °C. The pellet was discarded, and the supernatant was treated with 5 U/mL of Pierce Universal Nuclease (Fisher, 88,702) and incubated at 37 °C for 30 min. The solution was then centrifuged at 20,000 ×g for 30 min at 4 °C and the remaining supernatant was filtered using a 22 μm PES filter (Fisher, 595–3320). An iodixanol gradient was prepared in a 38.5 mL QuickSeal ultracentrifuge tube (Beckman Coulter, 344,326) by layering 8 mL of 15%, 6 mL of 25%, 4 mL of 40%, and 5 mL of 60% iodixanol solutions. All gradients were made using OptiPrep (Sigma, D1556) diluted with appropriate buffers. The 15% layer was prepared by mixing 5 mL of OptiPrep with 15 mL of 1 M NaCl/PBS-MK buffer (comprised of 200 µL 1 M KCl, 270 µL 1 M MgCl₂, and 20 mL 5 M NaCl in 80 mL PBS). The 25% layer contained 6.25 mL of OptiPrep, 8.75 mL of 1x PBS-MK (200 µL 1 M KCl and 270 µL 1 M MgCl₂ in 100 mL PBS), and 37.5 µL of phenol red. The 40% solution was prepared by mixing 8 mL of OptiPrep with 4 mL of 1× PBS-MK. The 60% iodixanol layer was prepared by mixing 12 mL of OptiPrep with 54 µL of phenol red. The viral lysate was gently loaded on top of the 60% gradient, and the remaining volume was filled with 1x PBS before heat-sealing the tubes. The tubes were placed in a Beckman Ti-70 rotor and ultracentrifuged at 200,000 ×g for 2 hours at 18 °C. The visible AAV-containing band found between the 40 and 60% layers was extracted using an 18-gauge needle. The virus was diluted tenfold in PBS and concentrated using an Amicon Ultra-15100 kDa centrifugal filter unit (Fisher, UFC810024), that was previously pre-treated with HBSS containing 5% Tween-20 for 3 hours. The virus was washed five times with 1xPBS at 1500 ×g, leaving a final volume of 500–800 μL that was supplemented with 0.001% Pluronic acid and stored at 80 °C until it was titrated.

Following a previously established protocol, both AAV6.2FF and AAV9 vectors were purified and DNA extracted using the Qiagen Blood and Tissue Kit (Qiagen 69,504, Germantown, Maryland) and the genome titers were determined using TaqMan quantitative polymerase chain reaction (qPCR) [36] The qPCR reaction contained a SV40-specific forward primer (5’-AGCAATAGCATCACAAATTTCACAA-3’), a reverse primer (5’CCAGACATGATAAGATACATTGATGAGTT-3’), and a fluorescent probe (/56-FAM/AGCATTTTT/Zen/TTCACTGCATTCTAGTTGTGGTTTGTC/3I ABkFQ)(Integrated DNA Technologies, Coralville, IA) mixed with Luna Universal qPCR Master Mix (New England Biolabs, Cat# M3003X, Ipswich, MA) and run on a LightCycler 480 thermocycler (Roche Nutly, New Jersey).

### SDS PAGE electrophoresis and coomassie blue staining

4x10^10^ vg of each vector sample was aliquoted and mixed with 4x reducing SDS sample buffer (Sigma-Aldrich, Oakville, ON, Canada) and denatured at 95 °C for 5 min. The samples were loaded onto a 6–15% SDS-polyacrylamide gradient gel and electrophoresed at 120 V for 1 hour. Following separation, the gel was stained in 0.1% Coomassie Brilliant Blue R-250 in 50% methanol and 10% glacial acetic acid on a shaker for 20 min and destained overnight in 40% methanol and 10% glacial acetic acid. The stained gel was imaged using the Chemidoc XRS imaging system (Bio-Rad, Hercules, CA, USA).

### Alkaline gel electrophoresis

2x10^10^ vg of each vector was mixed with 2x denaturing loading dye (2X Ficoll loading Dye-50655, 1 M NaOH, 2 mM EDTA, and 0.6% SDS) and denatured at 95 °C for 10 min. Samples were loaded onto a 1% agarose gel that was pre-soaked in 1x denaturing buffer (0.05 M NaOH, 1 mM EDTA) for 30 min. The gel was electrophoresed at 40 V for 14 hours overnight at 4 °C in the same 1x denaturing buffer. After the run, the gel was soaked in 1X TAE for 40 min and subsequently stained in 1X TAE containing SYBR Gold nucleic acid stain (ThermoFisher, Cat# S11494, Waltham, MA, USA) for 1 hour. The AAV genome was visualized using a UV transilluminator. Alkaline gel electrophoresis confirmed the presence of intact, full-length AAV genomes for all promoter constructs, with no evidence of truncation or genome instability (See Figs. S1 and S2).

### Animal experiments

Male C57BL/6 mice were purchased from Charles River Laboratories (Saint Constant, QC) at five weeks of age. They were given a one-week acclimatization period prior to any experimental procedures. Mice were randomized into experimental groups as groups of four and an additional two mice were assigned to each group as a positive control and a negative control. AAV6.2FF vectors driving expression from different putative tissue-specific promoters were delivered via intramuscular, intranasal, and intraperitoneal administrations at 1 × 10^11^ vector genomes (vg) each route. The AAV was diluted in sterile 1x PBS (Fisher A1 286,301, Massachusetts, United States) into final volumes of 40 µL, 80 µL, and 250 µL for IM, IN and IP administration routes, respectively. For every promoter group, an additional mouse was dosed with AAV6.2FF-AP, driven by the ubiquitous CASI promoter, serving as a positive control. For the promoters that were truly tissue-specific under these conditions, additional mice (*n* = 4) received AAV9-AP vectors driven by the identified tissue-specific promoter at 1 × 10^1 1^ vg to compare promoter activity between different serotypes. For any promoters that showed minimal off-target expression but strong transgene expression within the intended tissue, an additional group of mice (*n* = 4) received the same construct but via IP injection only at a dose of 1 × 10^1 1^ vg. At 21 days post AAV administration, mice were euthanized for tissue collection. The major organs harvested included the brain, heart, kidneys, liver, lungs, muscle, pancreas, and spleen. The lungs were perfused with 1xPBS via the right ventricle to blanch the lungs, after which the superior and post-caval lobes were removed and perfused with 5 mL of 2% paraformaldehyde using a 27-gauge needle and then placed into 2% paraformaldehyde to be fixed. The remaining three lung lobes were snap frozen in liquid nitrogen for downstream analysis. The non-pulmonary organs were sectioned into two representative tissue samples and either fixed in 2% paraformaldehyde and stained for AP expression macroscopically and microscopically, or flash-frozen for downstream homogenization and quantification of enzymatic AP activity assay.

### Macroscopic and microscopic analysis of alkaline phosphatase expression

The tissue samples previously fixed in paraformaldehyde were paraffin-embedded and sliced at a thickness of 5 µm onto Superfrost Plus microscope slides (Fisher 12–550-15, Pittsburgh, Pennsylvania). The slides underwent deparaffinization in xylene (3 ×5 min) and were then rehydrated through graded ethanol steps, including 100% ethanol (2 ×2 min), 90% ethanol (2 ×2 min), and 70% ethanol (1 ×2 min). After rehydration, the slides were equilibrated in alkaline phosphatase (AP) buffer (100 mM Tris-HCl, pH 8.5, 100 mM NaCl, 50 mM MgCl₂) for 5 min. The slides were AP stained overnight in the dark in a solution containing 100 mM Tris-HCl, pH 8.5, 100 mM NaCl, 50 mM MgCl2, 0.34 mg/ml nitroblue tetrazolium salt, 0.17 mg/ml X-Phos. The slides were then washed in 1xPBS twice for 2 min and counterstained with nuclear fast red (Sigma-Aldrich, N3020) for 1 minute. Excess stain was removed by rinsing with distilled water and then passed through a series of dehydration steps of 70%, 95%, 100% ethanol, with two dips each. The slides were then dried and dipped in xylene (2x 3 min) and cover-slipped with mounting medium (Tissue-Tek, 4583) and left overnight to dry. Brightfield images were taken at 10x magnification using an Olympus Accu-Scope EXC-400 microscope.

### Quantitative analysis of alkaline phosphatase expression

The tissue samples that were snap-frozen during necropsy were sectioned into 1 cm^3^ sections and transferred to screw cap tubes (MP Biomedicals 116,910,100) that were pre-loaded with a 1/4″ ceramic bead (MP Bio, 6,540,424) and TMNC buffer (50 mM Tris-HCl pH 7.5, 5 mM MgCl₂, 100 mM NaCl, 4% CHAPS). The samples were homogenized using a Precellys 24 tissue homogenizer (Thermo Fisher Scientific, Waltham, MA, USA) on program 4 (6000 rpm, 2 times for 1 min). The homogenized samples were heat-inactivated at 65 °C for 1 hour and spun at 14,000 rpm for 15 min at 4 °C. The homogenate was collected and stored at −80 °C for downstream quantitative analysis. The protein content of each sample was quantified using a Bradford assay. Briefly, each sample and bovine serum albumin (BSA) standards were mixed with 200 µL of 1x Coomassie Blue Plus Protein Assay Reagent (Thermo Fisher, 23,236) and loaded into a 96-well plate. Absorbance was measured at 595 nm using a Promega GloMax plate reader, and the protein concentrations were calculated from a BSA standard curve. To assess the amount of AP expressed in each sample, an enzymatic AP activity assay was performed. Human placental alkaline phosphatase (Sigma P3895) was diluted in a range of 10 ng/mL-400 ng/mL as the standard curve. Tissue homogenates were normalized to 30ug of protein and brought to a volume of 100 uL with TMNC buffer (50 mM Tris-HCl pH 7.5, 5 mM MgCl₂, 100 mM NaCl, 4% CHAPS) in black 96-well plates (Greiner Bio-One 655,900). A reaction master mix made up of 100 µL of 2x SEAP buffer (2 M diethanolamine, 1 mM MgCl₂, 10 mM L-homoarginine) and 5 µL of 4-methylumbelliferyl phosphate (MUP; Sigma M8883, 11.4 mg/mL in DMSO) was added to each well. Fluorescence was measured every 3 min for 1 hour at an excitation and emission of 355 nm and 440 nm, respectively, using the Promega GloMax plate reader. The enzymatic activity was calculated using the slope for each reaction and interpolating the values against the standard curve. The samples we then normalized for ng AP/mL/ug of protein.

### AAV genome quantification

Genomic DNA was isolated from paraffin-embedded (FFPE) tissue sections (8 in total) using the QIAamp DNA FFPE Tissue Kit (Qiagen 69,504, Germantown, MD, USA) following the manufacturer’s protocol, as all other tissue samples were fully allocated to tissue homogenization and enzymatic alkaline phosphatase activity assays with not enough sample for separate aliquots of high-quality genomic DNA. Quantification of vector genome copies was determined by qPCR using the same method previously outlined [36]. The concentration of DNA was determined using a NanoDrop spectrophotometer (Thermo Fisher Scientific), and vector genome copy numbers were normalized to total genomic DNA (vg/ng).

### Quantification and statistical analysis

All data analysis and graphs were performed using the GraphPad Prism 10 (San Diego, CA, USA) software. All graphs displayed individual biological replicates, with error bars representing the mean ± standard deviation. The data were compared using a one-way ANOVA followed by Tukey’s post hoc test for multiple comparisons, all assuming a Gaussian distribution. Statistical information, including sample size is indicated in the figure legends. Statistical significance is indicated as follows: *p ≤ 0.05, ** *p* ≤ 0.01, *** *p* ≤ 0.001, and **** *p* ≤ 0.0001.

## Results

### In vivo evaluation of algorithm-derived promoters identifies two skeletal muscle-specific and two cardiac-specific candidates for further testing

**Fig. 1:**
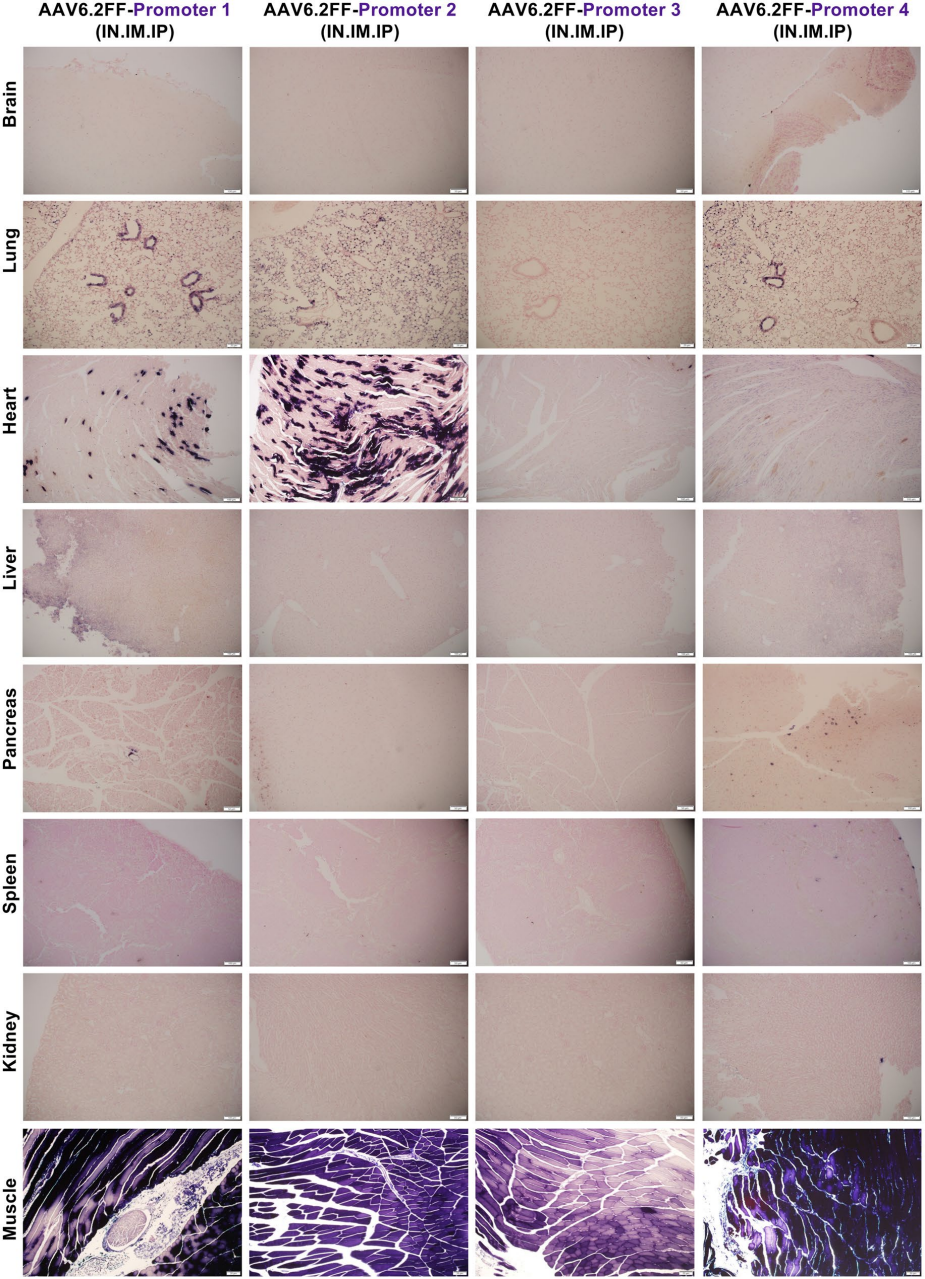
A microscopic view of AAV6.2FF-AP transduction driven by four algorithmically designed putative cardiac and skeletal muscle-specific promoters. AAV6.2FF expressing AP, under the control of four computationally derived and putative cardiac and skeletal muscle promoters (1, 2, 3 and 4), was administered to mice intranasally, intramuscularly, and intraperitoneally at a dose of 1 × 10^11^ vg per route. Three weeks following AAV administration, mice were euthanized, and tissues were collected, fixed, and stained for AP. Representative halves were paraffin-embedded, sectioned and restained for AP with nuclear fast red counterstaining to view exogenous alkaline phosphatase expression. Representative histological images captured at 10x magnification are shown

**Fig. 2:**
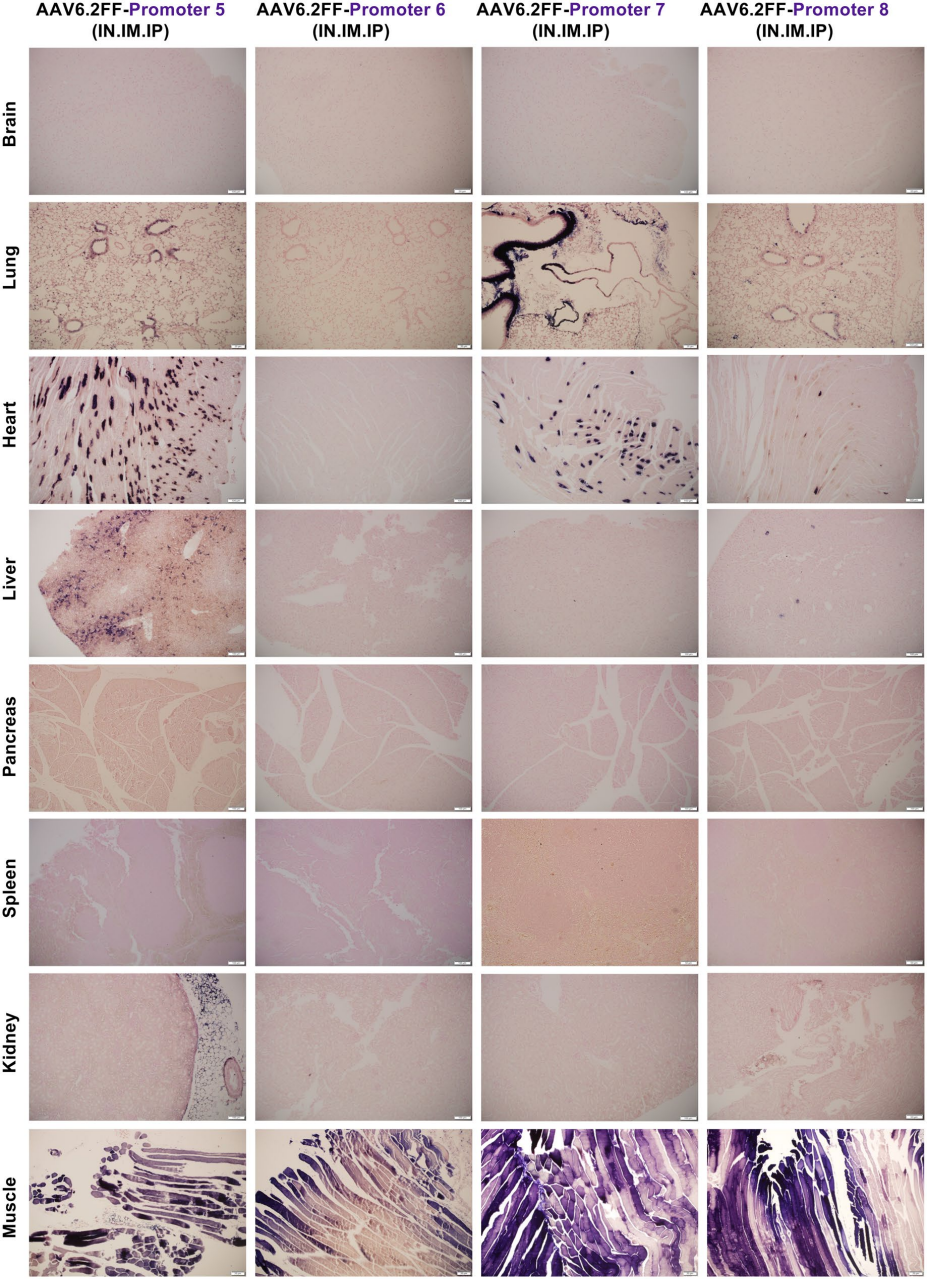
A microscopic view of AAV6.2FF-AP transduction driven by four algorithm-designed putative cardiac-specific promoters. AAV6.2FF expressing AP, under the control of four computationally derived and putative cardiac muscle promoters (5, 6, 7 and 8), was administered to mice intranasally, intramuscularly, and intraperitoneally at a dose of 1x10^11^ vg per route. Three weeks following AAV administration, mice were euthanized, and tissues were collected, fixed, and stained for AP. Representative halves were paraffin-embedded, sectioned and restained for AP with nuclear fast red counterstaining to view exogenous alkaline phosphatase expression. Representative histological images captured at 10x magnification are shown

**Fig. 3:**
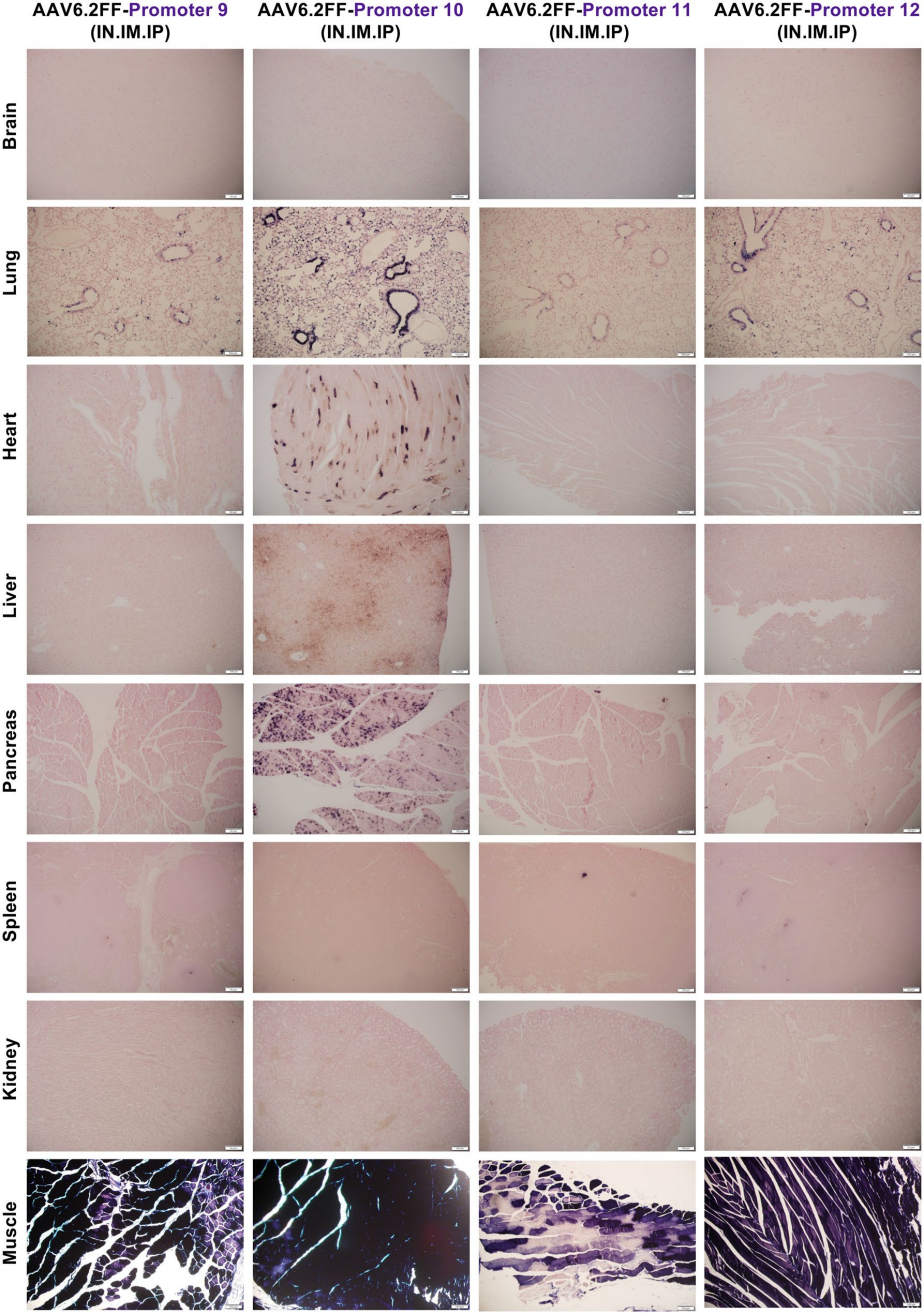
A microscopic view of AAV6.2FF-AP transduction driven by four algorithm-designed putative skeletal muscle-specific promoters. AAV6.2FF expressing AP, under the control of four computationally derived and putative skeletal muscle promoters (9, 10, 11 and 12), was administered to mice intranasally, intramuscularly, and intraperitoneally at a dose of 1x10^11^ vg per route. Three weeks following AAV administration, mice were euthanized, and tissues were collected, fixed, and stained for AP. Representative halves were paraffin-embedded, sectioned and restained for AP with nuclear fast red counterstaining to view exogenous alkaline phosphatase expression. Representative histological images captured at 10x magnification are shown

To evaluate how algorithm-derived tissue-specific promoters perform in vivo, we collaborated with Asimov (Boston, MA), who generated 12 synthetic promoter sequences with different predicted specificities using their proprietary algorithm. These promoters were designed using genomic data from both humans and mice to select regulatory sequences that drive gene expression in cardiac muscle, skeletal muscle, or both tissues simultaneously. Each promoter was cloned upstream of the human placental alkaline phosphatase reporter gene (AP) and packaged into the AAV6.2FF capsid for in vivo testing. AAV6.2FF-AP vectors containing algorithm-derived promoters were administered to C57BL/6 mice (*n* = 4 per promoter) via IN, IM, and IP injection at a dose of 1x10^11^ vector genome (vg) per route. Mice receiving AAV6.2FF-AP driven by the ubiquitous CASI promoter served as a positive control and mice receiving vehicle alone served as negative controls. Mice were euthanized 21 days post vector administration, and the brain, heart, lung, kidney, liver, pancreas, spleen and skeletal muscle were harvested and examined for AP expression through macroscopic (Figs. S3–S14) and microscopic (Figs. 1, 2 and 3) analysis. The 12 promoters were grouped (*n* = 4) according to their predicted tissue specificity as follows: promoters 1–4 were expected to drive expression in both the cardiac and skeletal muscle, promoters 5–8 were predicted to drive expression in cardiac muscle, and promoters 9–12 were predicted to drive expression in skeletal muscle.

Analysis of promoters 1–4 revealed that none of the promoters displayed exclusive activity in cardiac and skeletal muscle as initially predicted (Fig. 1, Figs. S3–S6). However, promoter 2 was unique in that it drove intense AP expression in the heart and skeletal muscle, while being completely inactive in the liver (Fig. 1, Fig. S5). Although some AP staining was observed in the lungs, the overall transgene expression pattern made promoter 2 a strong candidate for further analysis. Therefore, this promoter was selected for additional studies to explore whether systemic administration of AAV via IP injection alone would improve cardiac specificity and reduce the off-target expression in the lungs. While promoter 3 did not drive expression in both cardiac and skeletal muscle as initially predicted, it did demonstrate strong skeletal muscle specificity (Fig. 1, Fig. S5). No off-target expression was observed in any other tissues, even though the vector was delivered via all three routes of administration. Thus, promoter 3 was selected for subsequent testing using AAV9 to evaluate whether its specificity was maintained across different capsids.

When evaluating AP staining driven by promoters 5–8, none appeared to drive the cardiac-specific expression as expected (Fig. 2, Figs. S7–S10). Interestingly, promoter 6 consistently mediated strong expression in the skeletal muscle with no visible staining in any other tissues, including the liver, when delivered by all three routes (IN, IM and IP) (Fig. 2, Fig. S8). Similarly to promoter 3, we selected this promoter for subsequent testing using AAV9 to evaluate its specificity across different capsids. Promoter 7 stood out for its ability to express in the heart while completely silent the liver (Fig. 2, Fig. S9). Some off-target expression was observed in the lungs and skeletal muscle, but this was likely due to the direct administration via IN and IM routes. We decided to re-test promoter 7 using only IP administration, as was previously done with promoter 2.

Finally, we assessed promoters 9–12, which were all computationally predicted to be skeletal muscle specific (Fig. 3, Figs. S11–S14). While each promoter drove AP expression in the skeletal muscle, they were all associated with varying degrees of off-target activity and as a result, none of these promoters were selected for additional testing.

### Quantitative evaluation confirms the activity and specificity of the top cardiac and skeletal muscle-specific promoter candidates

**Fig. 4:**
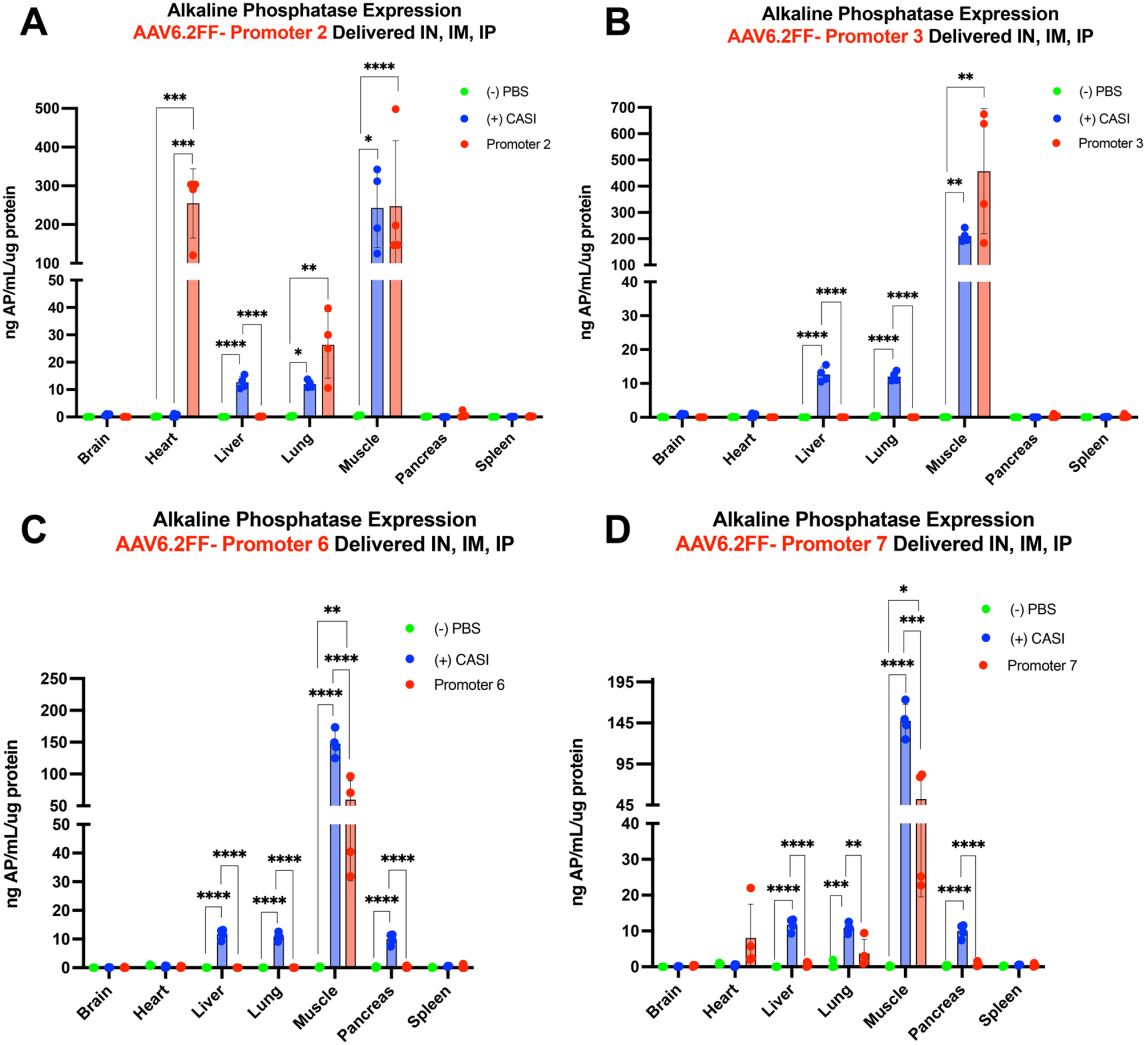
Quantitative alkaline phosphatase expression following administration of AAV6.2FF-AP from promoters 2, 3, 6 and 7. Mice received AAV6.2FF-AP driven by promoters 2 (**A**), 3 (**B**), 6 (**C**), and 7 (**D**), intranasally, intramuscularly and intraperitoneally at a dose of 1x10^11^ vg for each route of administration. Positive and negative control groups received AAV6.2FF-CASI-AP and PBS, respectively. Mice were euthanized 21 days post-AAV administration, tissues were collected, and a portion of each tissue was homogenized. Alkaline phosphatase expression was quantified across tissues using an enzymatic AP activity assay. Individual one-way ANOVAs were performed to compare the groups within each tissue. *p ≤ 0.05, ** *p* ≤ 0.01, *** *p* ≤ 0.001, and **** *p* ≤ 0.0001

**Fig. 5:**
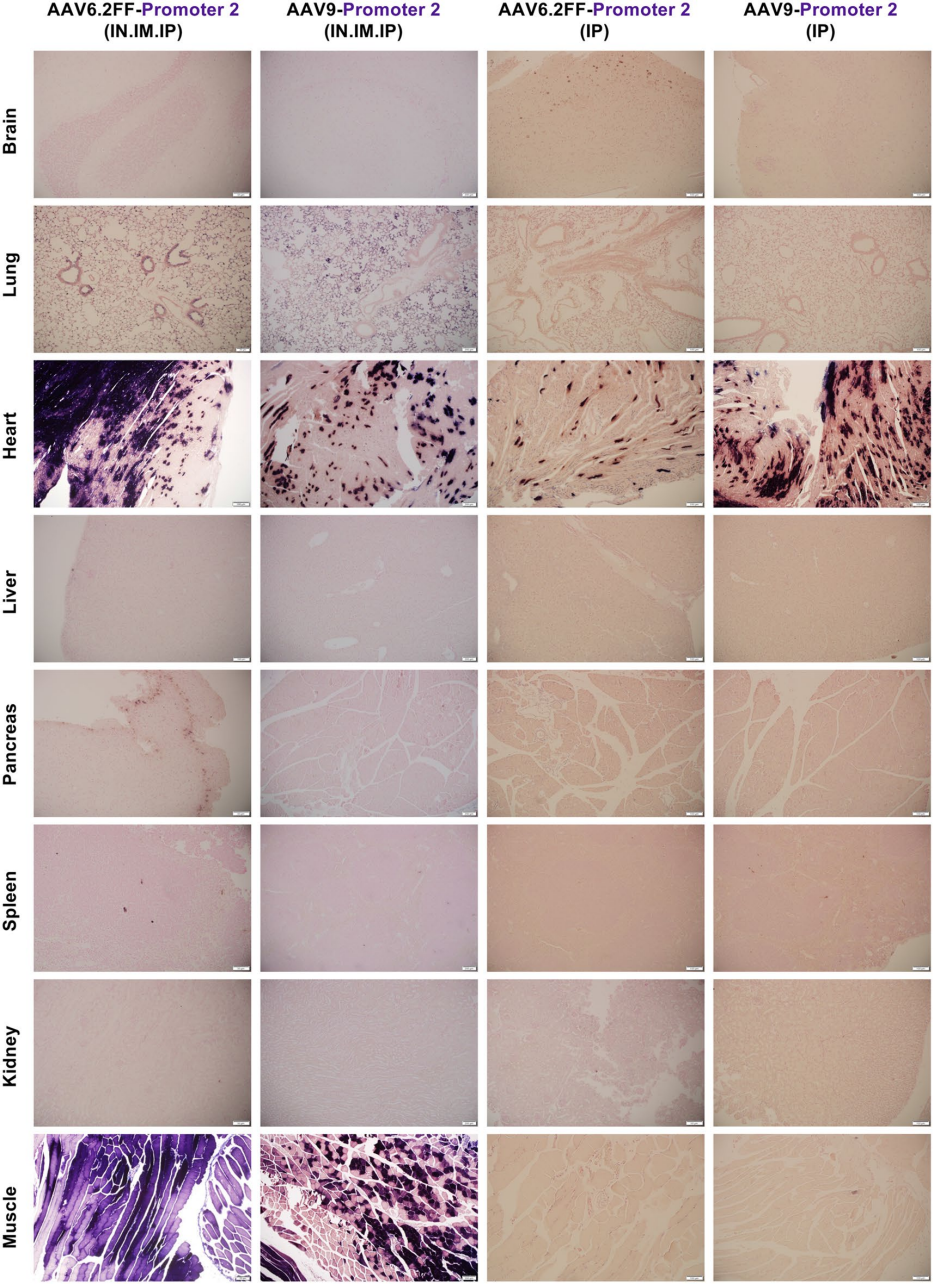
Histological analysis of AAV-mediated AP expression driven by promoter 2 across different AAV serotypes and delivery routes. Mice received AAV6.2FF-AP or AAV9-AP driven by promoter 2 administered via either combined routes (intranasal, intramuscular, and intraperitoneal) or a single intraperitoneal injection at a dose of 1x10^11^ vg per route. Mice were euthanized 21 days post AAV administration, and tissues were collected, fixed and stained for AP. Tissues were subsequently embedded in paraffin, sectioned and stained to detect AP expression, with nuclei counterstained using nuclear fast red. Representative images taken at 10x magnification are shown

To quantify the AP expression in tissues harvested from transduced mice, we performed an enzymatic alkaline phosphatase activity assay for all promoter groups. A representative piece of each tissue was collected during necropsy and homogenized in lysis buffer. A Bradford assay was used to determine the protein concentration of each tissue lysate. Based on the protein concentration, AP activity was measured for each tissue and normalized for total protein, displayed as ng AP/mL per µg protein (Figs. 4 and 5, Fig. S15). Of the 12 promoters examined, promoter 2 drove the highest AP expression in the heart with a mean activity of 254.56 ng AP/mL/µg protein and significantly outperformed the CASI promoter (*p* = 0.0002) (Fig. 4A). Promoter 3 was among the top three promoters that drove the strongest AP activity in the skeletal muscle but stood out for its ability to de-target the liver and all other tissues (Fig. 4B) Although the difference was not statistically significant, promoter 3 showed a higher mean activity in the muscle compared to the CASI promoter, with a mean of 456.99 ng AP/mL per µg protein and 211.77 ng AP/mL per µg protein, respectively (Fig. 4B). Promoter 6 was also skeletal muscle-specific, as seen by the baseline levels in all other tissues, including the liver (Fig. 4C). On the contrary, promoter 7 did show off-target AP expression in the heart, lung, and skeletal muscle (Fig. 4D). However, although not statistically significant, its stronger AP expression in the heart compared to CASI suggests its potential as a cardiac-specific promoter with further refinement. Overall, promoters 2, 3, 6 and 7 were identified as the most promising candidates chosen for follow-up studies to confirm their tissue specificity using different delivery routes or AAV capsids.

### Heart-specific expression from promoters 2 and 7 achieved via intraperitoneal delivery across AAV capsids

Promoters 2 and 7 were previously identified as having the potential to be cardiac-specific, based on their strong AP activity, which displayed higher levels than the broadly active CASI promoter. However, when administered via all three routes (IN, IM, and IP), both promoters showed modest off-target AP expression in tissues such as the lungs and skeletal muscle. To determine whether transgene expression could be confined more specifically to the heart, we re-evaluated promoters 2 and 7 using IP administration alone, using both AAV6.2FF and AAV9 capsids.

**Fig. 6:**
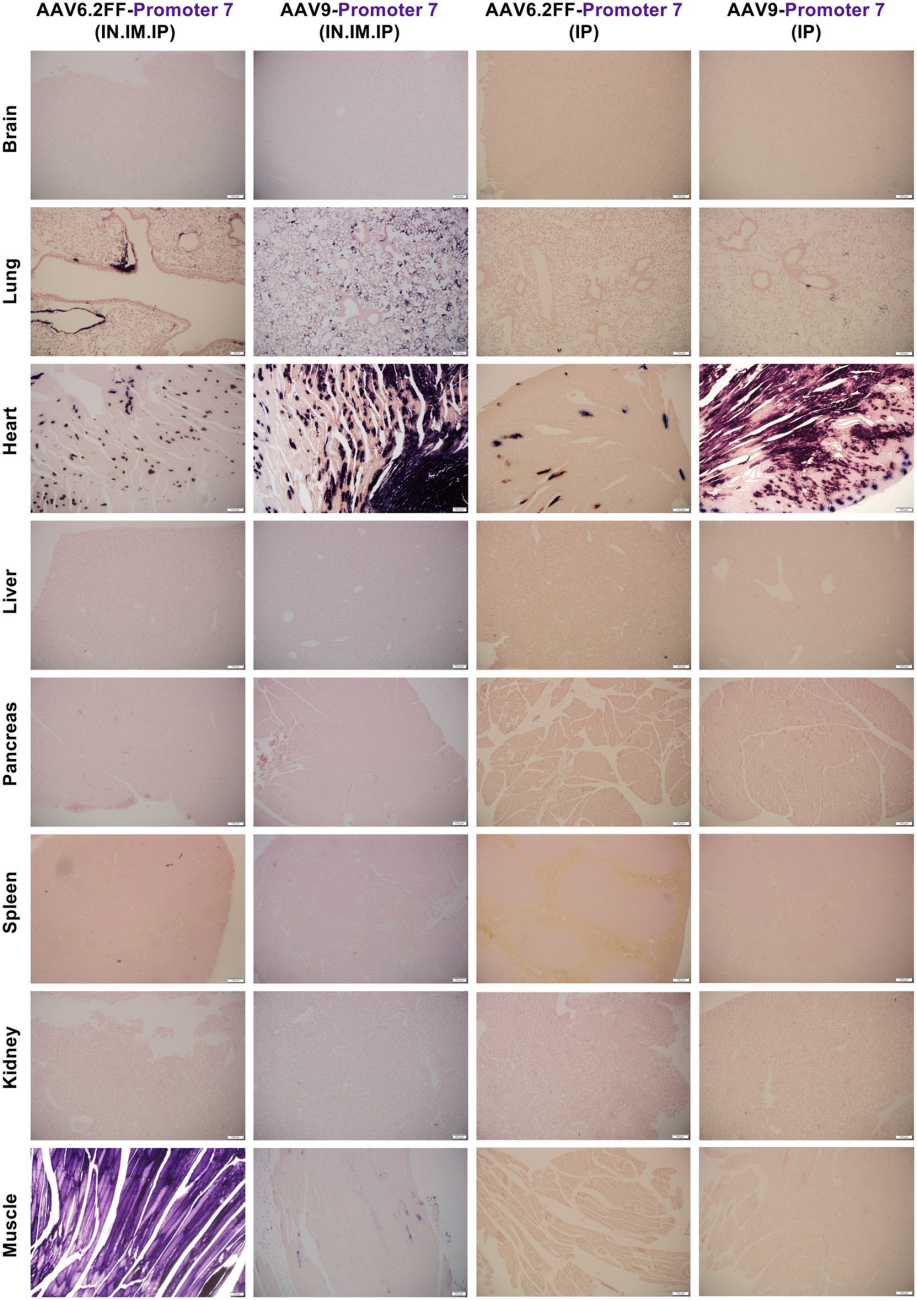
Histological analysis of AAV-mediated AP expression driven by promoter 7 across different AAV serotypes and different routes of administration. Mice received AAV6.2FF-AP or AAV9-AP driven by promoter 7 administered via either combined routes (intranasal, intramuscular, and intraperitoneal) or a single intraperitoneal injection at a dose of 1x10^11^ vg per route. Mice were euthanized 21 days post AAV administration, and tissues were collected, fixed and stained for AP. Tissues were subsequently embedded in paraffin, sectioned and stained to detect AP expression, with nuclei counterstained using nuclear fast red. Representative images taken at 10x magnification are shown

**Fig. 7:**
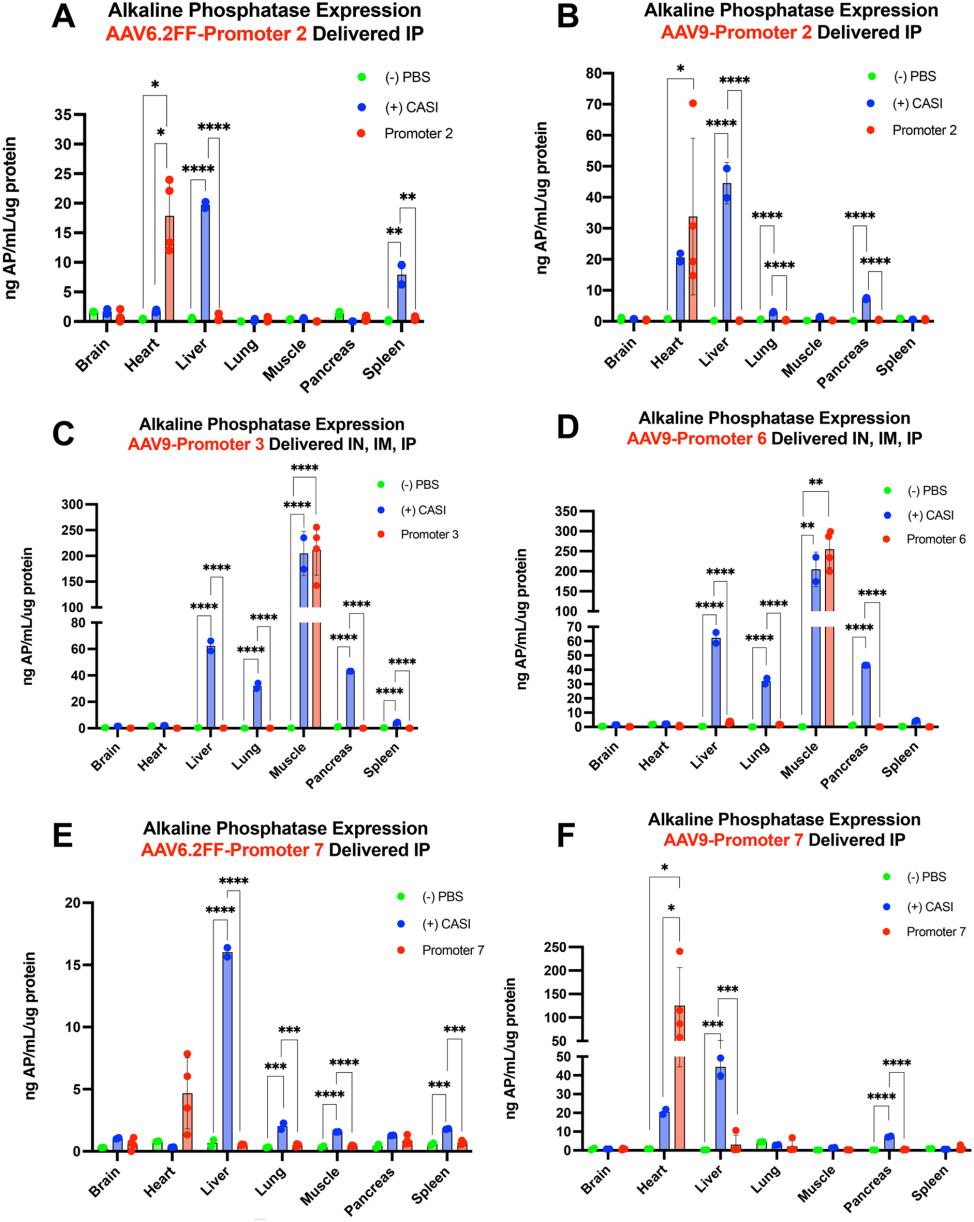
Quantitative analysis of tissue-wide AAV-mediated AP expression driven by promoters 2, 3, 6 and 7 across different AAV serotypes and different routes of administration. Mice were administered AAV vectors encoding AP under the control of either promoters 2, 3, 6, 7 or CASI. AAV6.2FF or AAV9 vectors were delivered at a dose of 1 × 10^1 1^ vg per route using one of the following strategies: (**A**) AAV6.2FF-Promoter 2-AP via intraperitoneal injection only; (**B**) AAV9-promoter 2-AP via intraperitoneal injection only; (**C**) AAV9-promoter 3-AP via combined intranasal, intramuscular, and intraperitoneal routes at 1 × 10^1 1^ vg per route; (**D**) AAV9-promoter 6-AP via combined intranasal, intramuscular, and intraperitoneal routes at 1 × 10^1 1^ vg per route; (**E**) AAV6.2FF-Promoter 7-AP via intraperitoneal injection only; or (**F**) AAV9-promoter 7-AP via intraperitoneal injection only. Mice were euthanized three weeks post-AAV administration, and a portion of each tissue was homogenized. Alkaline phosphatase expression was quantified across tissues using an enzymatic AP activity assay. Individual one-way ANOVAs were performed to compare the groups within each tissue. *p ≤ 0.05, ** *p* ≤ 0.01, *** *p* ≤ 0.001, and **** *p* ≤ 0.0001

Macroscopic (Figs. S16–S17) and microscopic (Fig. 5) AP staining confirmed that promoter 2, when delivered via the IP route alone restricted AP expression to the heart, with no visible staining in non-cardiac tissues. This pattern was consistent across both AAV6.2FF and AAV9 capsids, indicating that the route of administration alone was sufficient to eliminate off-target expression (Fig. 5). Similarly, promoter 7 was also heart-specific when delivered via IP administration, effectively de-targeting all other tissues across both capsids (Fig. 6, Figs. S18–S19). Importantly, promoters 2 and 7 showed complete de-targeting of the liver, a common site for off-target expression for systemic delivery. In contrast, the CASI promoter targeted the liver regardless of being delivered IP, or with the AAV9 capsid (Fig. S20). When paired with AAV9, promoters 2 and 7 achieved higher AP expression in the heart with means of 33.79 ng AP/mL per µg protein and 125.23 ng AP/mL per µg protein, respectively (Figs. 7B and 7F). Under these conditions, promoter 7 statistically outperformed the CASI promoter (*p* = 0.0275) (Fig. 7F). These findings suggest that using a single systemic delivery route and avoiding the direct vector administration to the lungs and skeletal muscle can effectively restrict transgene expression to the cardiac tissue.

### Skeletal muscle-specific promoters 3 and 6 retain their specificity across AAV capsids

**Fig. 8:**
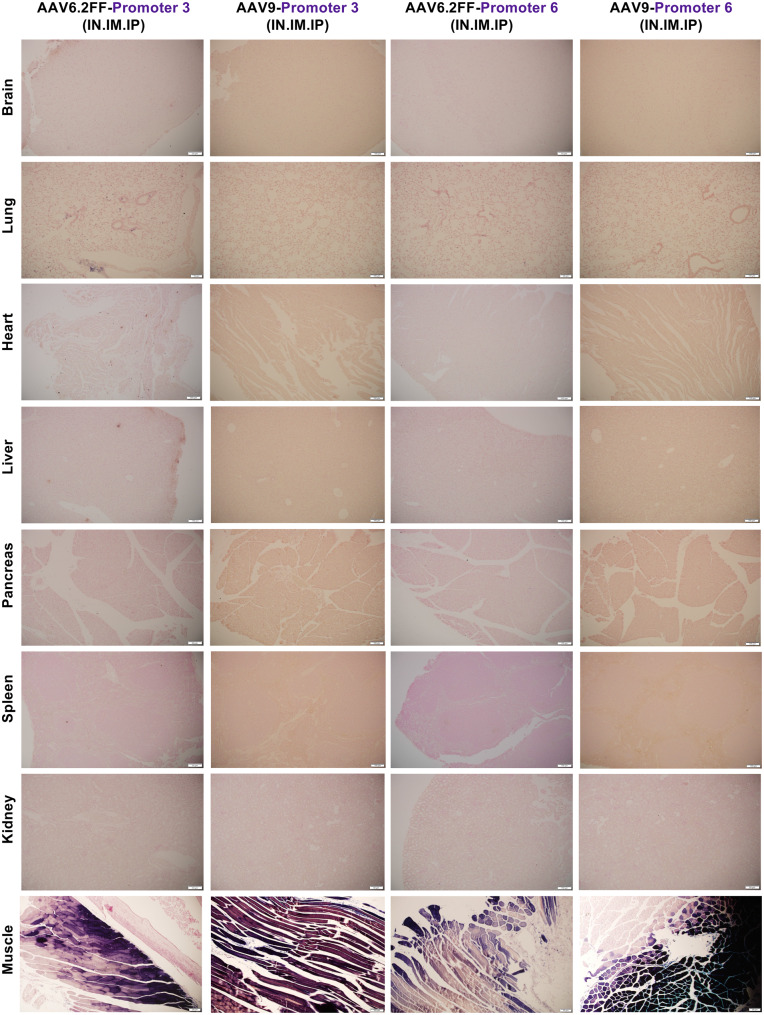
Histological analysis of AAV-mediated AP expression driven by promoters 3 and 6 across different AAV serotypes via IN, IM and IP administration. Mice received either AAV6.2FF-AP or AAV9-AP driven by either promoter 3 or 6, administered via combined routes (intranasal, intramuscular, and intraperitoneal) at a dose of 1 × 10^^11^ vg per route. Mice were euthanized 21 days post AAV administration, and tissues were collected, fixed and stained for AP. Tissues were subsequently embedded in paraffin, sectioned and stained to detect AP expression, with nuclei counterstained using nuclear fast red. Representative images taken at 10x magnification are shown

Since promoters 3 and 6 were already naturally muscle-specific when administered via all three routes with AAV6.2FF, we wanted to determine whether AAV capsid selection affects promoter performance and specificity. Therefore, we re-evaluated the promoters using AAV9 via the same triple-route administrations and AP analysis as outlined above. Regardless of the AAV capsid used, both promoters mediated restricted skeletal muscle expression while completely de-targeting the liver and all other tissues as demonstrated both microscopically (Fig. 8) and macroscopically (Figs. S21–S23). Promoter 6 showed a clear boost in skeletal muscle expression when delivered with AAV9, increasing from 59.77 ng of AP per mL per µg of protein with AAV6.2FF to 254.89 ng of AP per mL per µg of protein with AAV9. These findings show that promoters 3 and 6 are truly skeletal muscle-specific regardless of the capsid type or delivery route used and that switching capsids can be an effective way to increase expression levels without broadening tissue distribution.

### Vector genome biodistribution analysis confirms cardiac and skeletal muscle restriction is promoter-driven

To confirm that the transgene expression we observed with promoters 2,3, 6 and 7 was due to the promoter specificity and not the lack of vector delivery to certain tissues, we quantified AAV vector genome copies in all major organs. This analysis allowed us to assess vector genome biodistribution independent of AP expression. DNA was extracted from paraffin-embedded tissue sections, vector genome levels were measured using qPCR and normalized to total genomic DNA (vg/ng). All values were background-corrected by subtracting the vector genome copies found in PBS-treated controls and presented in Fig. S23. Across all promoters and capsids tested, vector genomes were detected in multiple tissues confirming that AAV vectors successfully reached sites beyond the intended tissue target but did not express. In addition, vector genomes were detected in the liver for all promoter constructs, confirming that the absence of AP expression in this tissue was due to lack of promoter activity, rather than ineffective vector delivery (Fig. S23). As expected, the mice that received vector via all three routes (IN, IM, and IP) showed higher overall vector genome distribution compared to the ones that received the IP route alone, likely reflecting the difference in total vector dose (3x10^11^ vg vs 1x10^11^ vg, respectively). Despite the widespread vector genome distribution, transgene expression remained tightly restricted to the heart or the skeletal muscle, depending on the promoter confirming that the expression patterns are due to promoter specificity and not limited biodistribution.

## Discussion

As gene therapy continues to advance toward precision medicine, the need for tighter spatial control over transgene expression is becoming increasingly important. Although many AAV-based platforms have demonstrated success using strong ubiquitous promoters, their broad activity profile increases the risk of various immune responses, off-target toxicity and transgene silencing [6, 23]. To overcome these limitations, we evaluated a panel of 12 computationally-derived tissue-specific promoters predicted to restrict AAV-mediated transgene expression to either the cardiac muscle, skeletal muscle or both muscles. Alkaline gel electrophoresis confirmed the presence of intact, full-length AAV genomes for all promoter constructs, with no evidence of truncation or genome instability. We evaluated every promoter for strength and specificity in vivo using AAV6.2FF to deliver an AP reporter gene and subsequently assessed the influence of capsid choice or route of administration on the most promising candidates.

When selecting promoters for further evaluation, we prioritized those that showed three critical characteristics: tight tissue specificity, strong transgene expression ideally exceeding the expression levels of the broadly active CASI promoter, and complete liver de-targeting. The liver tends to accumulate a disproportionate number of AAV vector genomes following systemic administration which can lead to unintended transgene expression and immune responses that can compromise therapeutic efficacy [12, 37, 38]. Therefore, identifying promoters that actively silence transgene expression in the liver while driving strong expression in the target tissues would be an important advancement for gene therapies that require high doses or systemic administration.

From our initial screen, we identified four promising promoters: two with strong transgene expression in the cardiac muscle (promoters 2 and 7), and two with skeletal muscle-restricted activity (promoters 3 and 6). None of them drove detectable transgene expression in the liver, even though AAV biodistribution quantification confirmed the presence of vector genomes in the liver for each promoter construct. This indicated that tissue specificity was conferred by promoter design and not because of limited vector biodistribution. When promoter constructs 2 and 7 were delivered via all three routes (IN, IM, and IP), both promoters showed strong expression in the heart but also in the lungs and skeletal muscle, likely due to the higher vector retention and cellular uptake associated with local delivery [39]. Intranasal and intramuscular administrations provide direct exposure of the vector to epithelial and myofibrillar surfaces, compared to the biodistribution seen with systemic administrations [39–41]. Therefore, we repeated vector delivery of these two promoter constructs but this time only through the intraperitoneal route to avoid direct lung and muscle exposure. Under these delivery conditions, both promoters showed exclusive and strong cardiac AP expression greater than the expression elicited from the CASI promoter. Promoter 7 significantly outperformed the CASI promoter when delivered with AAV9, suggesting that capsid choice is another element to enhance transgene expression. Promoters 3 and 6 were identified as exceptionally promising candidates because of their intrinsic specificity, which was maintained even when vectors were delivered locally via intranasal and intramuscular routes of administration. Unlike other promoters that required route refinement to achieve targeted transgene expression, promoters 3 and 6 demonstrated natural restriction to the skeletal muscle. When these promoters were re-evaluated using the AAV9 capsid known for broader tropism than AAV6.2FF, the promoters retained their specificity and continued to de-target the liver.

During our quantitative analysis, we unexpectedly measured high background AP activity in the kidneys of all mice and cohorts, including those treated with PBS. This AP activity was heat-stable and consistent with previously reported endogenous AP expression in mock-infected mice [41]. Because macroscopic and histological images showed no detectable transduction in the renal tissue, we excluded the quantitative values from all the graphs to avoid any misinterpretation.

Across all promoters and delivery conditions, we found the presence of AAV vector genomes in all tissues, including the liver, confirming that tissue specificity was in fact due to the promoter design and not ineffective vector delivery. As expected, the mice that received AAV via all three routes of administration had more vector genomes present across tissues than the mice that only received IP administration likely because they received a cumulative dose of 3x10^11^ vg. Remarkably, promoters 3 and 6 retained complete skeletal muscle specificity even with a higher vector load and systemic vector genome spread associated with the triple route delivery. Promoters with this level of precision hold great promise for treating monogenic skeletal muscle disorders such as Duchenne muscular dystrophy (DMD) and X-linked myotubular myopathy (XLMTM). Currently, ELEVIDYS is the only FDA-approved AAV gene therapy targeting skeletal muscle disease [42, 43]. It treats DMD and uses a synthetic muscle-specific promoter known as MHCK7 [42, 43]. Despite its muscle-targeted design, both preclinical and clinical studies have reported off-target expression in the liver, along with associated liver toxicity in some patients [44–46]. These findings show that even with tissue-specific promoters, systemic or high-dose delivery can lead to gene expression in unintended parts of the body. On the contrary, our data shows that promoters 3 and 6 drive exclusive skeletal muscle expression, even after systemic delivery via three delivery routes, representing a major step forward in addressing these concerns.

One limitation to our study design is that we assessed transgene expression only 21 days after vector administration and did not evaluate whether these promoters could maintain long-term expression or resist transgene silencing over time. Secondly, although we quantified AAV vector genomes in all major tissues, we did not quantify transgene mRNA levels, which could provide further insight into transduction mechanisms. Thirdly, with our relatively small sample size per promoter group (*n* = 4) it is possible that we could have missed subtle differences in expression or variability between animals. Future work should increase sample sizes, employ a trackable reporter gene such as secreted alkaline phosphatase (SEAP) to assess transgene expression over time, and include an RT-qPCR to quantify transgene mRNA levels. Lastly, while testing in human muscle cell lines is a valuable next step, we focused initially on in vivo evaluation because cell‑line models only partially capture the regulatory context that shapes promoter activity, and our promoter designs, built using both human and mouse genomic datasets, enabled us to narrow down the most promising candidates, which we now plan to validate in human systems moving forward.

## Conclusions

In summary, we have shown that computationally designed elements can outperform a broadly active promoter like CASI not only in targeting but also in terms of strength. We identified two naturally specific promoters that exclusively drive transgene expression in skeletal muscle under all delivery conditions and AAV capsid types. In addition, we discovered two promoters that only express in the heart when delivered intraperitoneally. While this study focuses exclusively on cardiac and skeletal muscle promoters, the broad implication is that algorithmically designed promoters can achieve remarkable tissue specificity, especially when matched with a suitable AAV capsid and route of administration. The ability to fine-tune the strength and localization of transgene expression expands the therapeutic potential of AAV vectors for a range of monogenic diseases.

## Electronic supplementary material

Below is the link to the electronic supplementary material.

Supplementary Material 1

## Data Availability

Due to intellectual property considerations, distribution of the promoter sequences and their associated plasmids is subject to certain restrictions. Requests for access and material transfer should be directed to Asimov, where they will be evaluated and, if appropriate, made available under a material transfer agreement (MTA).

## Abbreviations

AAV: Adeno-associated virus
Vg: Vector genomes
PBS: Phosphate-buffered saline
qPCR: Quantitative PCR
TF: Transcription factor
AP: Alkaline Phosphatase
IN: Intranasal
IM: Intramuscular
IP: Intraperitoneal
CASI: CMV enhancer + chicken β-actin promoter + intron
CMV: Cytomegalovirus
CAG: Chimeric chicken-β-actin promoter made up from the cytomegalovirus immediate early (CMV) promoter, chimeric chicken-β-actin (CBA), and ubiquitin C (UBC) enhancer region
