# Supplementary material for: Identification of computationally designed skeletal and cardiac promoters with high specificity across AAV delivery routes
Source: BMC Biotechnol. 2026 Mar 26;26:60. doi: 10.1186/s12896-026-01141-1 (PMC13147773; doi:10.1186/s12896-026-01141-1)
Supplement: Supplementary file 1 — Supplementary Material 1 [file 12896_2026_1141_MOESM1_ESM.pdf]

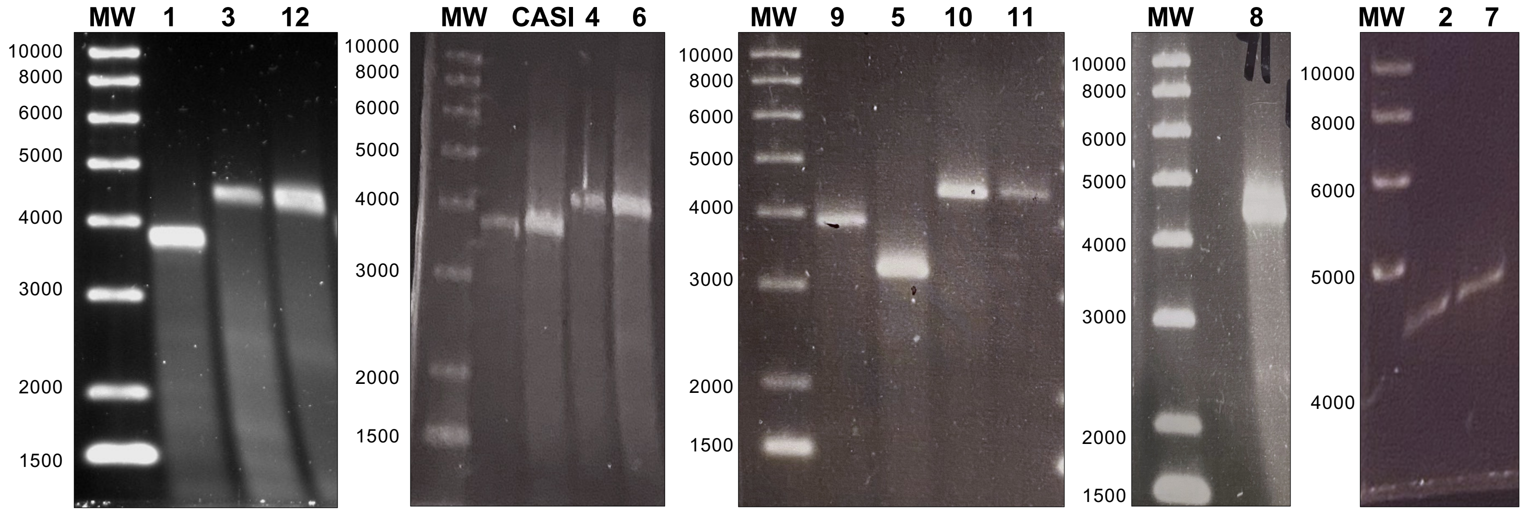

Figure S1. Analysis of AAV6.2FF vector genome integrity by alkaline gel electrophoresis. To evaluate AAV genome integrity of our AAV6.2FF vector stocks, AAV vector samples (between $1×10^{10}$ vg and $4×10^{10}$ vg per lane) were denatured in alkaline loading buffer and resolved on a 1% agarose gel under alkaline conditions (NaOH/EDTA) to assess vector genome integrity. Gels were run at low voltage overnight at 4 °C, neutralized, and stained with SYBR Gold prior to imaging. Lanes are labelled according to promoter number. Banding patterns reflect the size and integrity of single-stranded vector genomes.

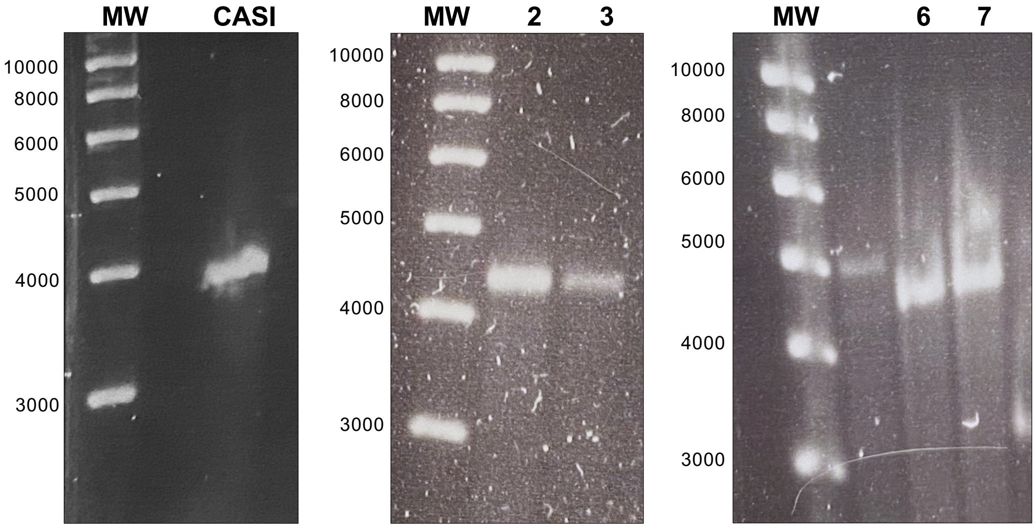

Figure S2. Analysis of AAV9 vector genome integrity by alkaline gel electrophoresis. To evaluate AAV genome integrity of our AAV9 vector stocks, AAV vector samples (between $1×10^{10}$ vg and $4×10^{10}$ vg per lane) were denatured in alkaline loading buffer and resolved on a 1% agarose gel under alkaline conditions (NaOH/EDTA) to assess vector genome integrity. Gels were run at low voltage overnight at 4 °C, neutralized, and stained with SYBR Gold prior to imaging. Lanes are labelled according to promoter number. Banding patterns reflect the size and integrity of single-stranded vector genomes.

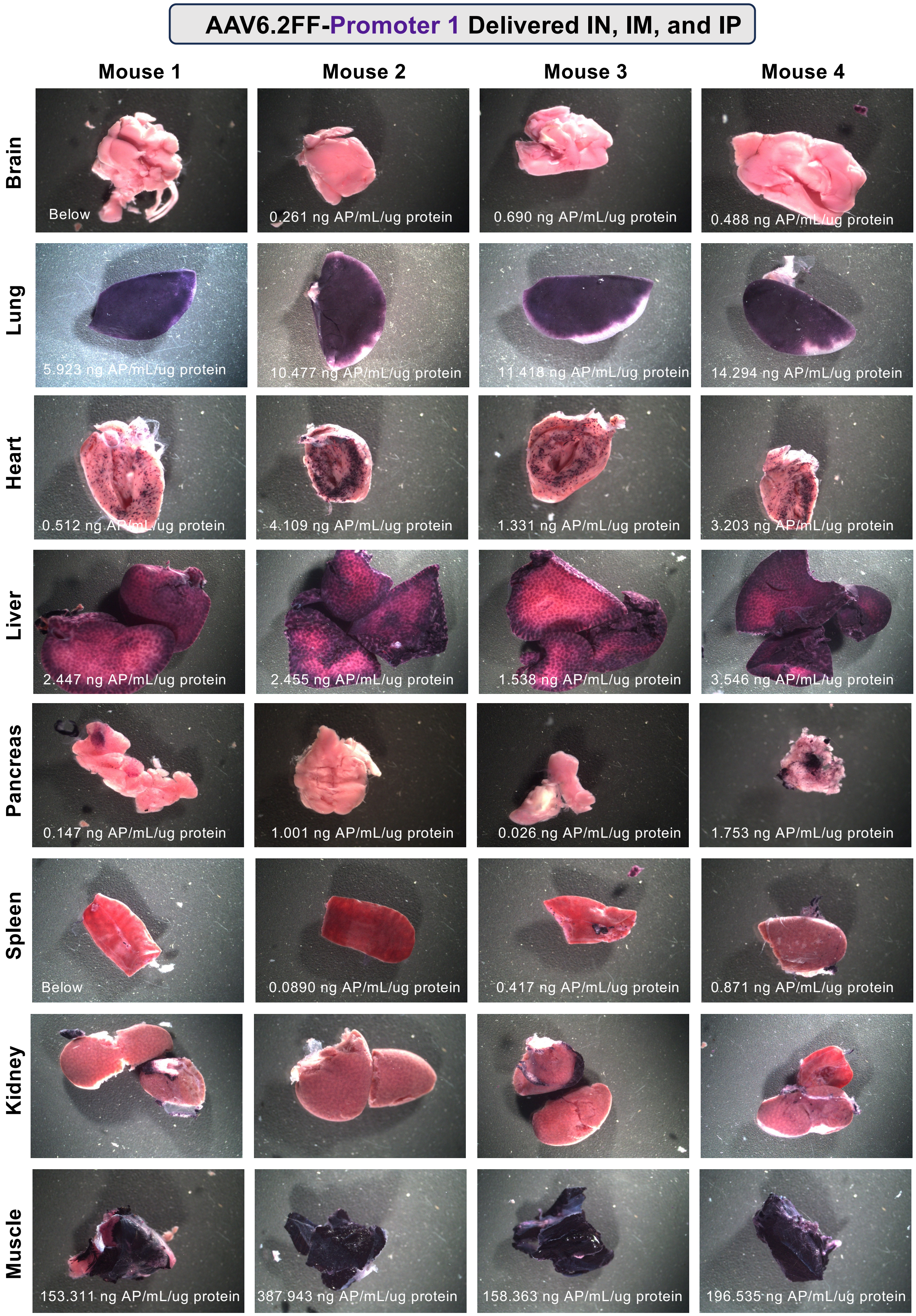

Figure S3. Tissue-wide macroscopic and quantitative analysis of AAV6.2FF-mediated AP expression driven by promoter 1 delivered intranasally, intramuscularly, and intraperitoneally. C57BL/6 mice received $1x10^{11}$ vg (per route) of AAV6.2FF expressing alkaline phosphatase (AP) driven by promoter 1 via intranasal (IN), intramuscular (IM), and intraperitoneal (IP) administration. At three weeks post-AAV administration, mice were euthanized, and tissues were collected for either macroscopic AP staining or quantitative AP activity analysis. Shown are gross images from all mice and corresponding AP expression levels (ng AP/mL/μg protein) for each tissue.

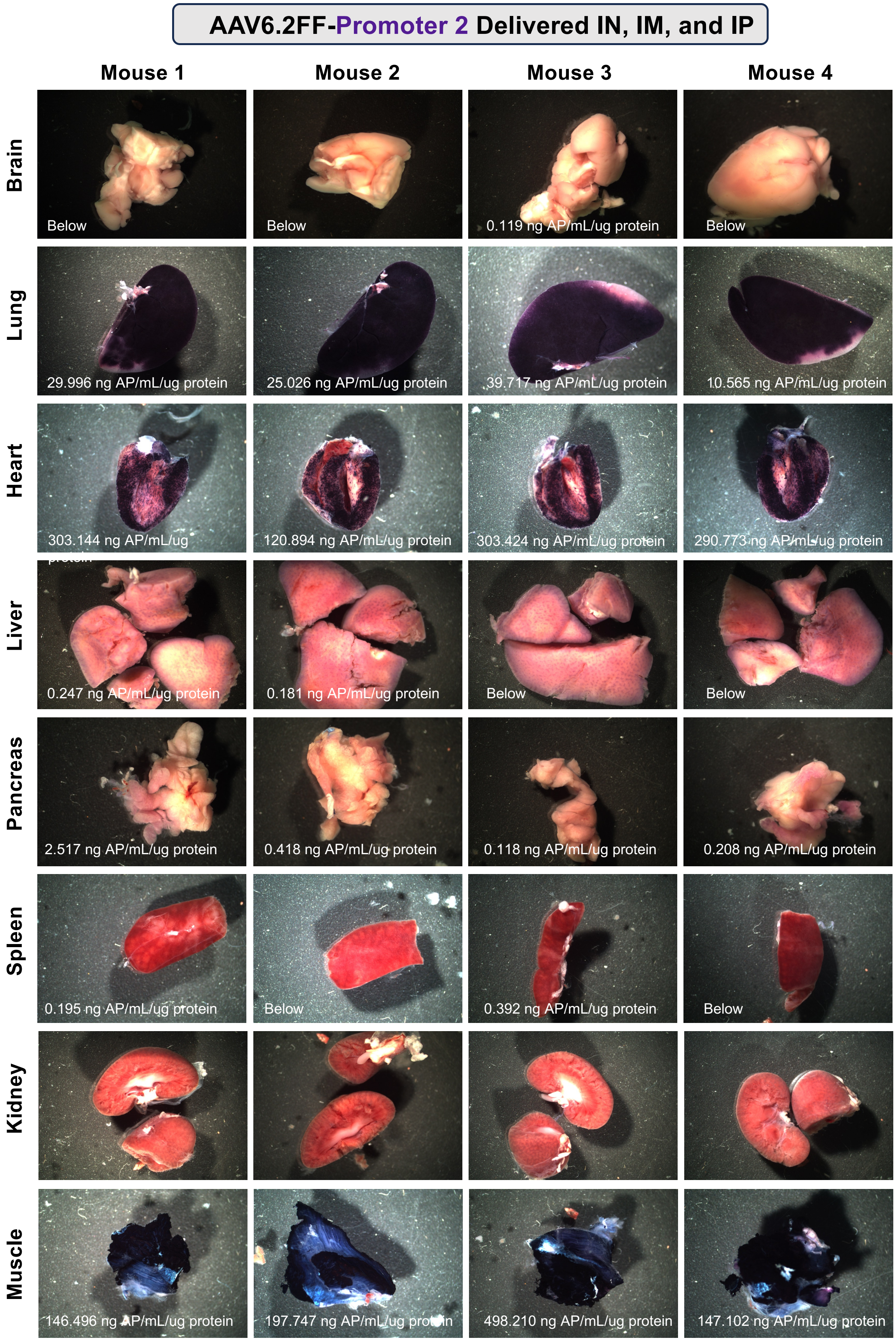

Figure S4. Tissue-wide macroscopic and quantitative analysis of AAV6.2FF-mediated AP expression driven by promoter 2 delivered intranasally, intramuscularly, and intraperitoneally. C57BL/6 mice received $1x10^{11}$ vg (per route) of AAV6.2FF expressing alkaline phosphatase (AP) driven by promoter 2 via intranasal (IN), intramuscular (IM), and intraperitoneal (IP) administration. At three weeks post-AAV administration, mice were euthanized, and tissues were collected for either macroscopic AP staining or quantitative AP activity analysis. Shown are gross images from all mice and corresponding AP expression levels (ng AP/mL/μg protein) for each tissue.

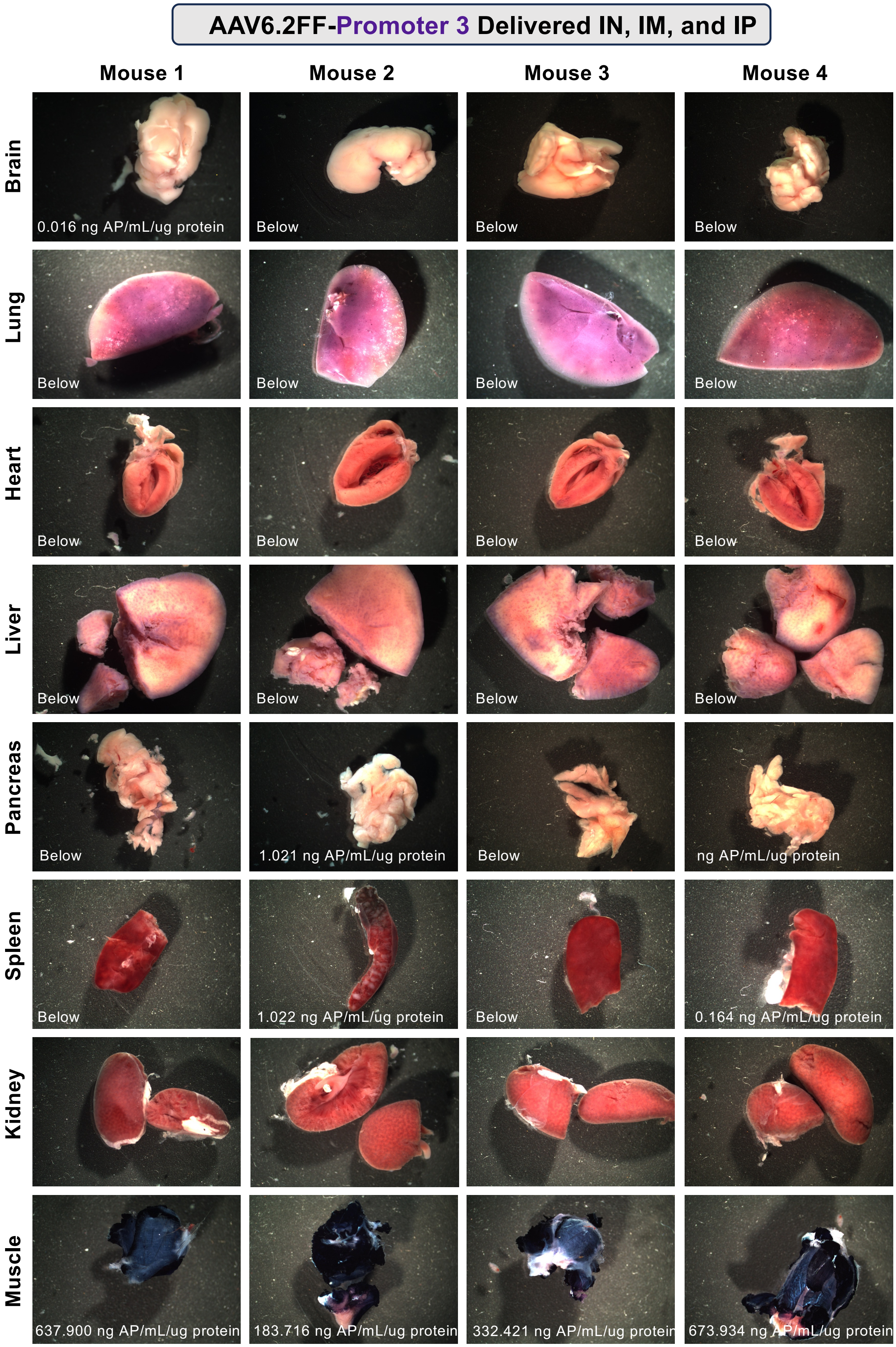

Figure S5. Tissue-wide macroscopic and quantitative analysis of AAV6.2FF-mediated AP expression driven by promoter 3 delivered intranasally, intramuscularly, and intraperitoneally. C57BL/6 mice received $1x10^{11}$ vg (per route) of AAV6.2FF expressing alkaline phosphatase (AP) driven by promoter 3 via intranasal (IN), intramuscular (IM), and intraperitoneal (IP) administration. At three weeks post-AAV administration, mice were euthanized, and tissues were collected for either macroscopic AP staining or quantitative AP activity analysis. Shown are gross images from all mice and corresponding AP expression levels (ng AP/mL/μg protein) for each tissue.

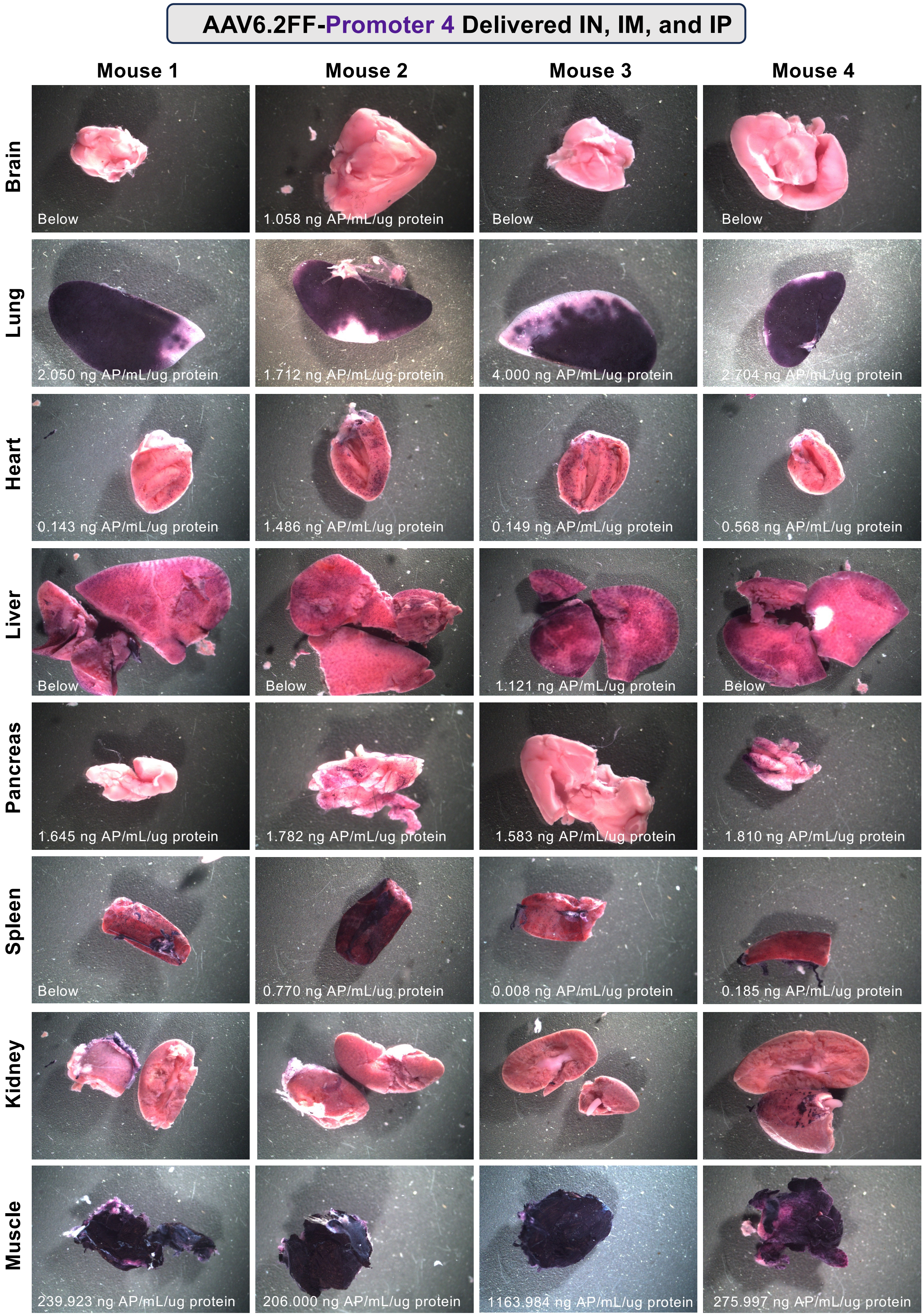

Figure S6. Tissue-wide macroscopic and quantitative analysis of AAV6.2FF-mediated AP expression driven by promoter 4 delivered intranasally, intramuscularly, and intraperitoneally. C57BL/6 mice received 1×10^{11} vg (per route) of AAV6.2FF expressing alkaline phosphatase (AP) driven by promoter 4 via intranasal (IN), intramuscular (IM), and intraperitoneal (IP) administration. At three weeks post-AAV administration, mice were euthanized, and tissues were collected for either macroscopic AP staining or quantitative AP activity analysis. Shown are gross images from all mice and corresponding AP expression levels (ng AP/mL/μg protein) for each tissue.

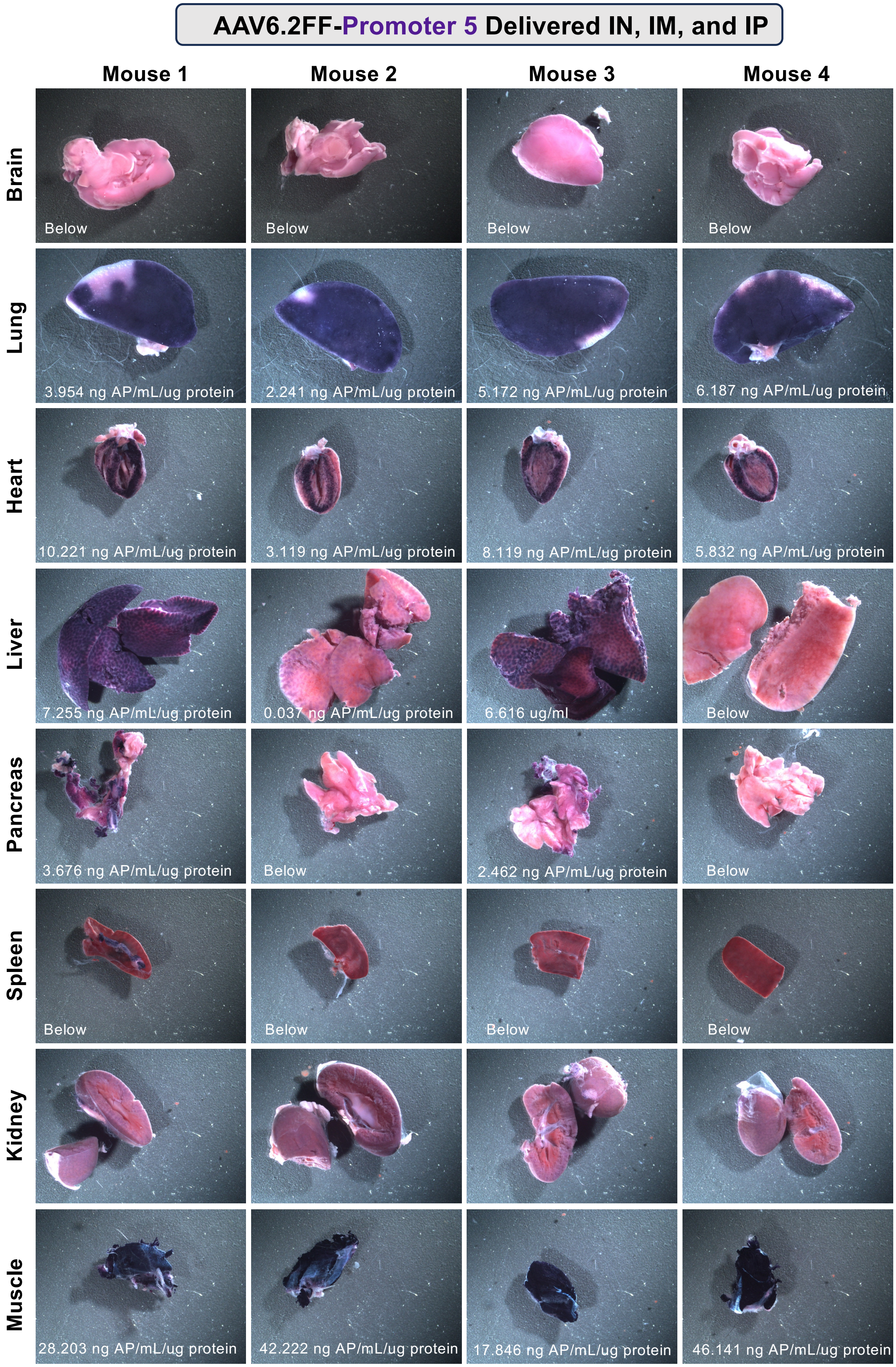

Figure S7. Tissue-wide macroscopic and quantitative analysis of AAV6.2FF-mediated AP expression driven by promoter 5 delivered intranasally, intramuscularly, and intraperitoneally. C57BL/6 mice received 1x10^{11} vg (per route) of AAV6.2FF expressing alkaline phosphatase (AP) driven by promoter 5 via intranasal (IN), intramuscular (IM), and intraperitoneal (IP) administration. At three weeks post-AAV administration, mice were euthanized, and tissues were collected for either macroscopic AP staining or quantitative AP activity analysis. Shown are gross images from all mice and corresponding AP expression levels (ng AP/mL/µg protein) for each tissue.

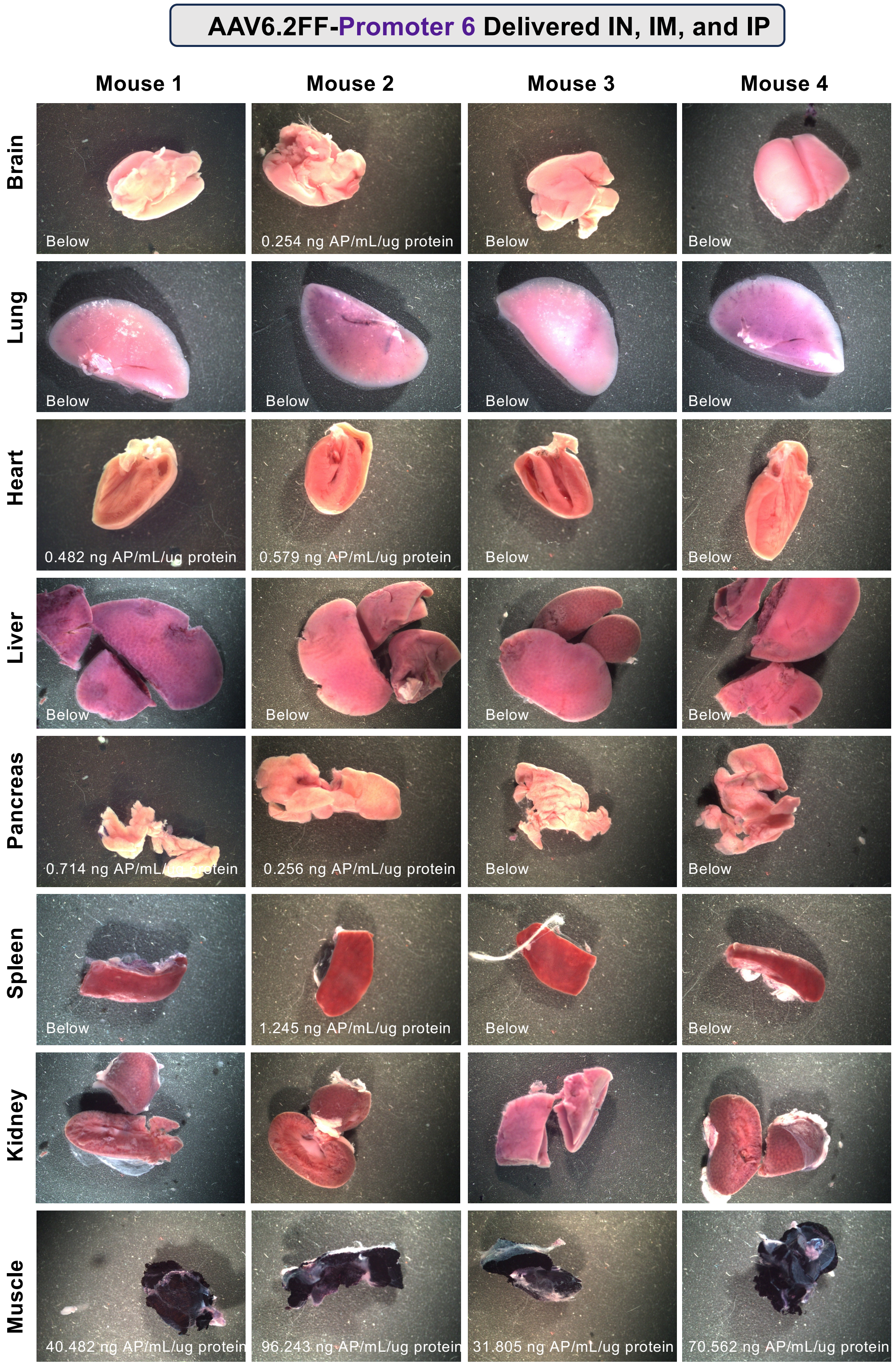

Figure S8. Tissue-wide macroscopic and quantitative analysis of AAV6.2FF-mediated AP expression driven by promoter 6 delivered intranasally, intramuscularly, and intraperitoneally. C57BL/6 mice received $1x10^{11}$ vg (per route) of AAV6.2FF expressing alkaline phosphatase (AP) driven by promoter 6 via intranasal (IN), intramuscular (IM), and intraperitoneal (IP) administration. At three weeks post-AAV administration, mice were euthanized, and tissues were collected for either macroscopic AP staining or quantitative AP activity analysis. Shown are gross images from all mice and corresponding AP expression levels (ng AP/mL/µg protein) for each tissue.

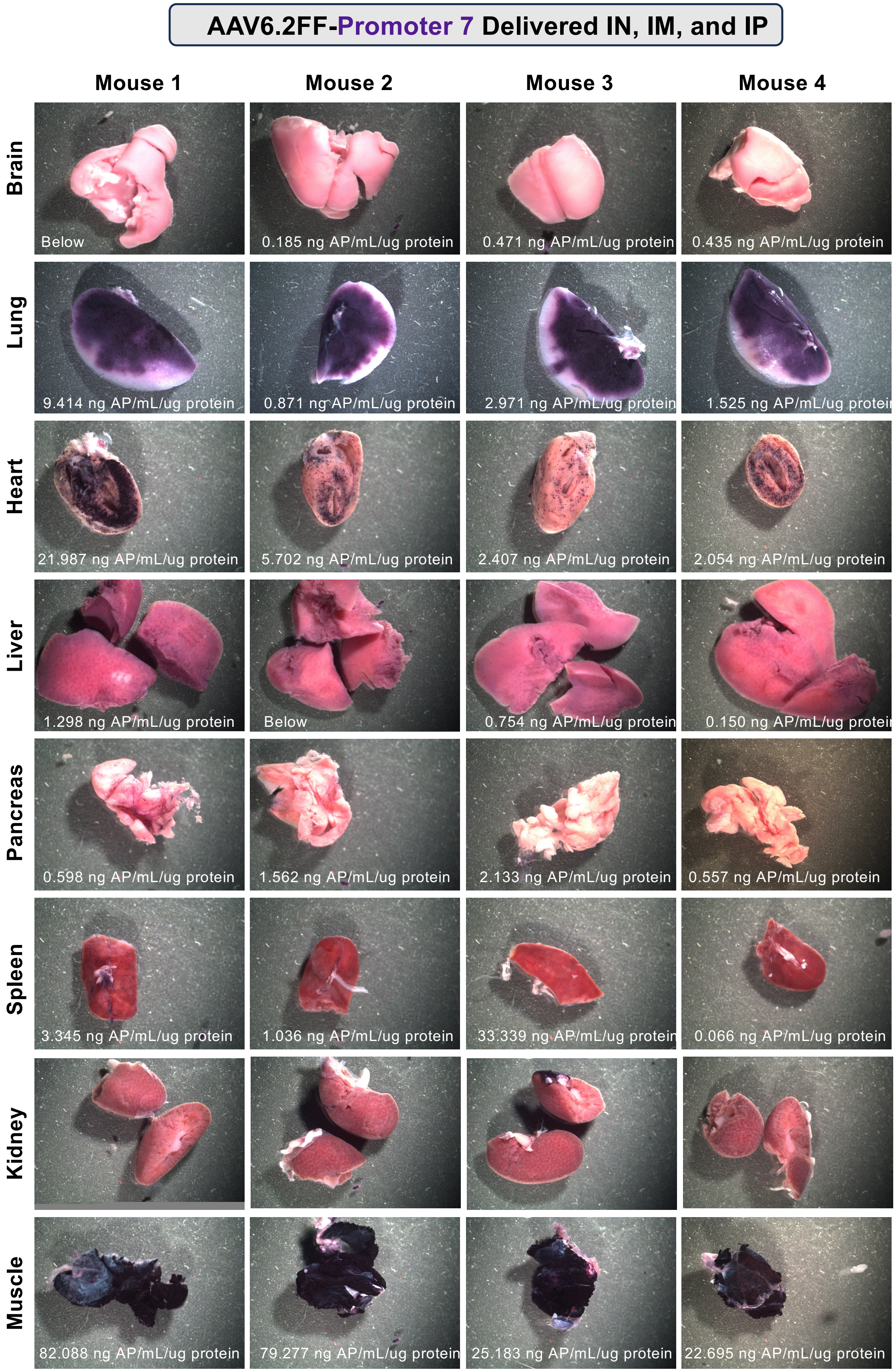

Figure S9. Tissue-wide macroscopic and quantitative analysis of AAV6.2FF-mediated AP expression driven by promoter 7 delivered intranasally, intramuscularly, and intraperitoneally. C57BL/6 mice received 1x10^{11} vg (per route) of AAV6.2FF expressing alkaline phosphatase (AP) driven by promoter 7 via intranasal (IN), intramuscular (IM), and intraperitoneal (IP) administration. At three weeks post-AAV administration, mice were euthanized, and tissues were collected for either macroscopic AP staining or quantitative AP activity analysis. Shown are gross images from all mice and corresponding AP expression levels (ng AP/mL/μg protein) for each tissue.

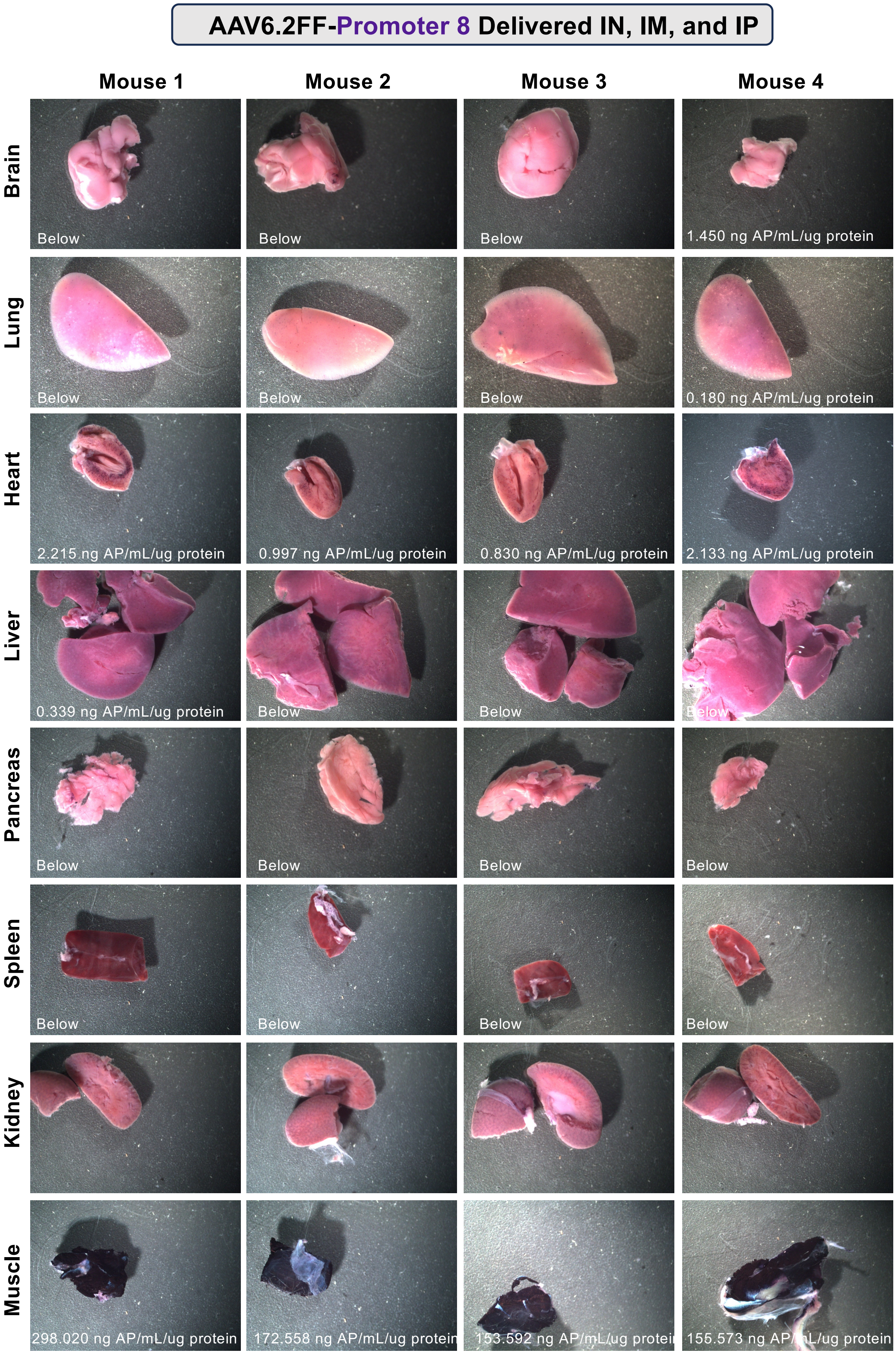

Figure S10. Tissue-wide macroscopic and quantitative analysis of AAV6.2FF-mediated AP expression driven by promoter 8 delivered intranasally, intramuscularly, and intraperitoneally. C57BL/6 mice received 1x10^{11} vg (per route) of AAV6.2FF expressing alkaline phosphatase (AP) driven by promoter 8 via intranasal (IN), intramuscular (IM), and intraperitoneal (IP) administration. At three weeks post-AAV administration, mice were euthanized, and tissues were collected for either macroscopic AP staining or quantitative AP activity analysis. Shown are gross images from all mice and corresponding AP expression levels (ng AP/mL/µg protein) for each tissue.

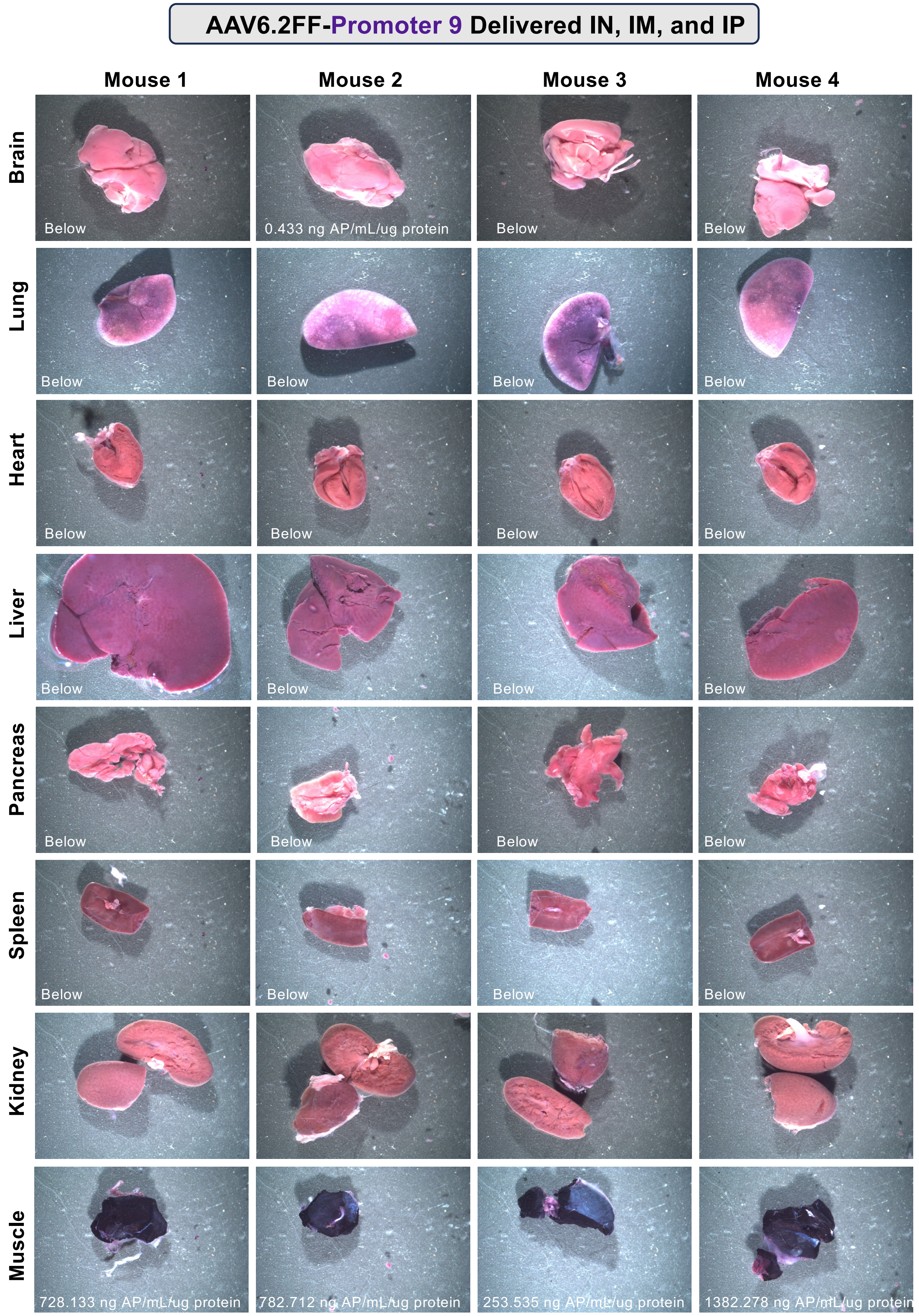

Figure S11. Tissue-wide macroscopic and quantitative analysis of AAV6.2FF-mediated AP expression driven by promoter 9 delivered intranasally, intramuscularly, and intraperitoneally. C57BL/6 mice received 1x10^{11} vg (per route) of AAV6.2FF expressing alkaline phosphatase (AP) driven by promoter 9 via intranasal (IN), intramuscular (IM), and intraperitoneal (IP) administration. At three weeks post-AAV administration, mice were euthanized, and tissues were collected for either macroscopic AP staining or quantitative AP activity analysis. Shown are gross images from all mice and corresponding AP expression levels (ng AP/mL/µg protein) for each tissue.

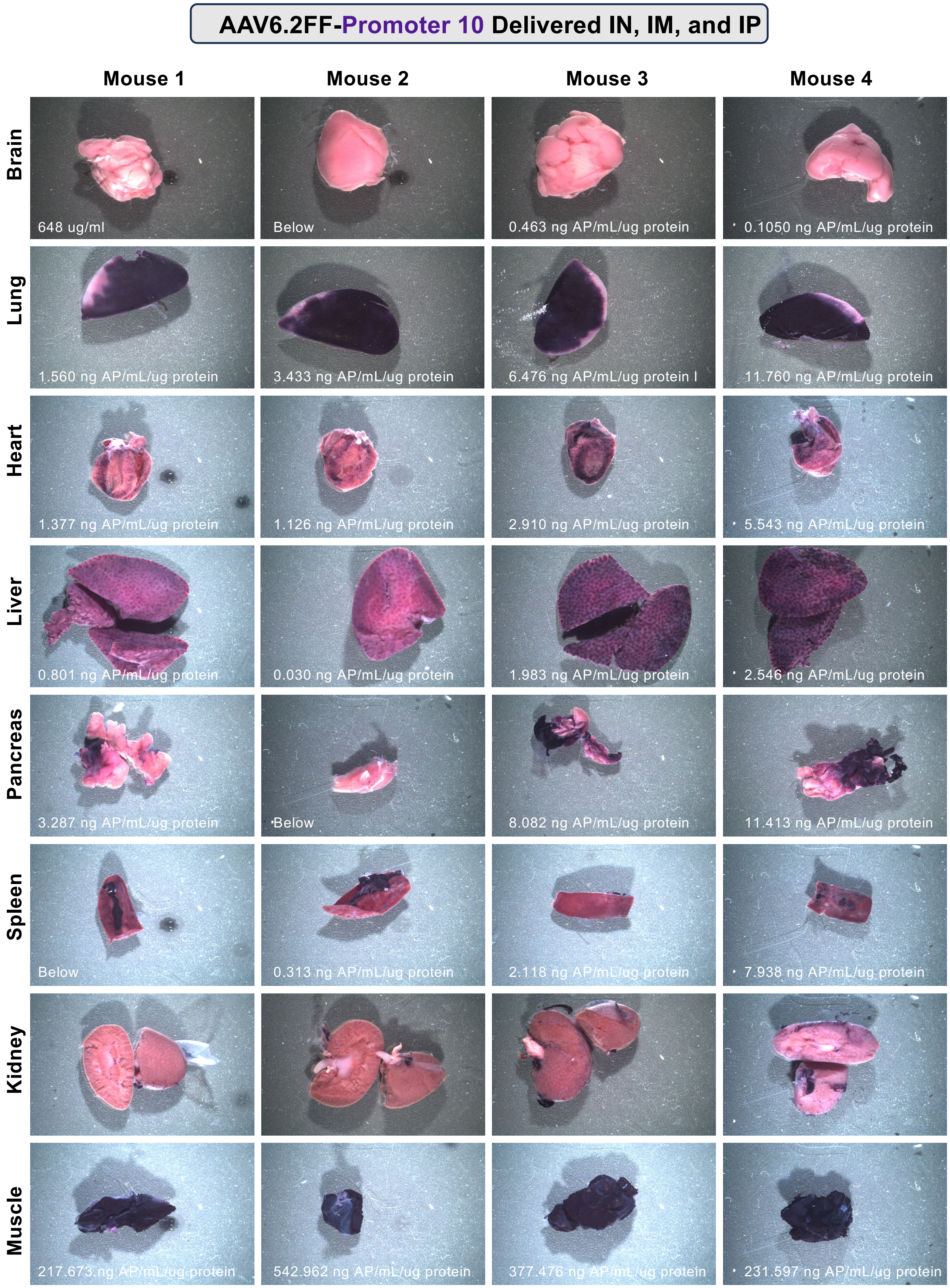

Figure S12. Tissue-wide macroscopic and quantitative analysis of AAV6.2FF-mediated AP expression driven by promoter 10 delivered intranasally, intramuscularly, and intraperitoneally. C57BL/6 mice received $1x10^{11}$ vg (per route) of AAV6.2FF expressing alkaline phosphatase (AP) driven by promoter 10 via intranasal (IN), intramuscular (IM), and intraperitoneal (IP) administration. At three weeks post-AAV administration, mice were euthanized, and tissues were collected for either macroscopic AP staining or quantitative AP activity analysis. Shown are gross images from all mice and corresponding AP expression levels (ng AP/mL/µg protein) for each tissue.

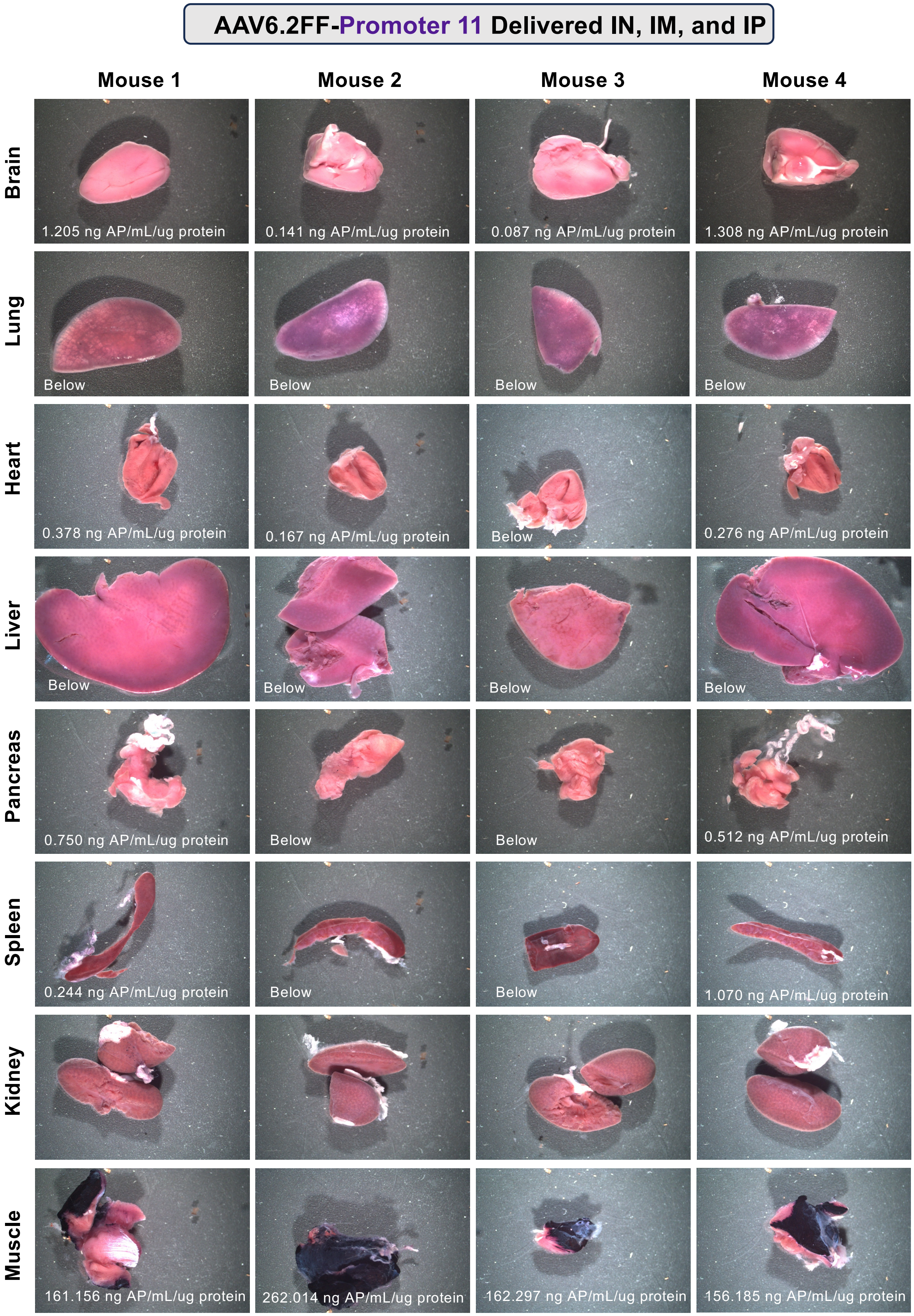

Figure S13. Tissue-wide macroscopic and quantitative analysis of AAV6.2FF-mediated AP expression driven by promoter 11 delivered intranasally, intramuscularly, and intraperitoneally. C57BL/6 mice received $1x10^{11}$ vg (per route) of AAV6.2FF expressing alkaline phosphatase (AP) driven by promoter 11 via intranasal (IN), intramuscular (IM), and intraperitoneal (IP) administration. At three weeks post-AAV administration, mice were euthanized, and tissues were collected for either macroscopic AP staining or quantitative AP activity analysis. Shown are gross images from all mice and corresponding AP expression levels (ng AP/mL/μg protein) for each tissue.

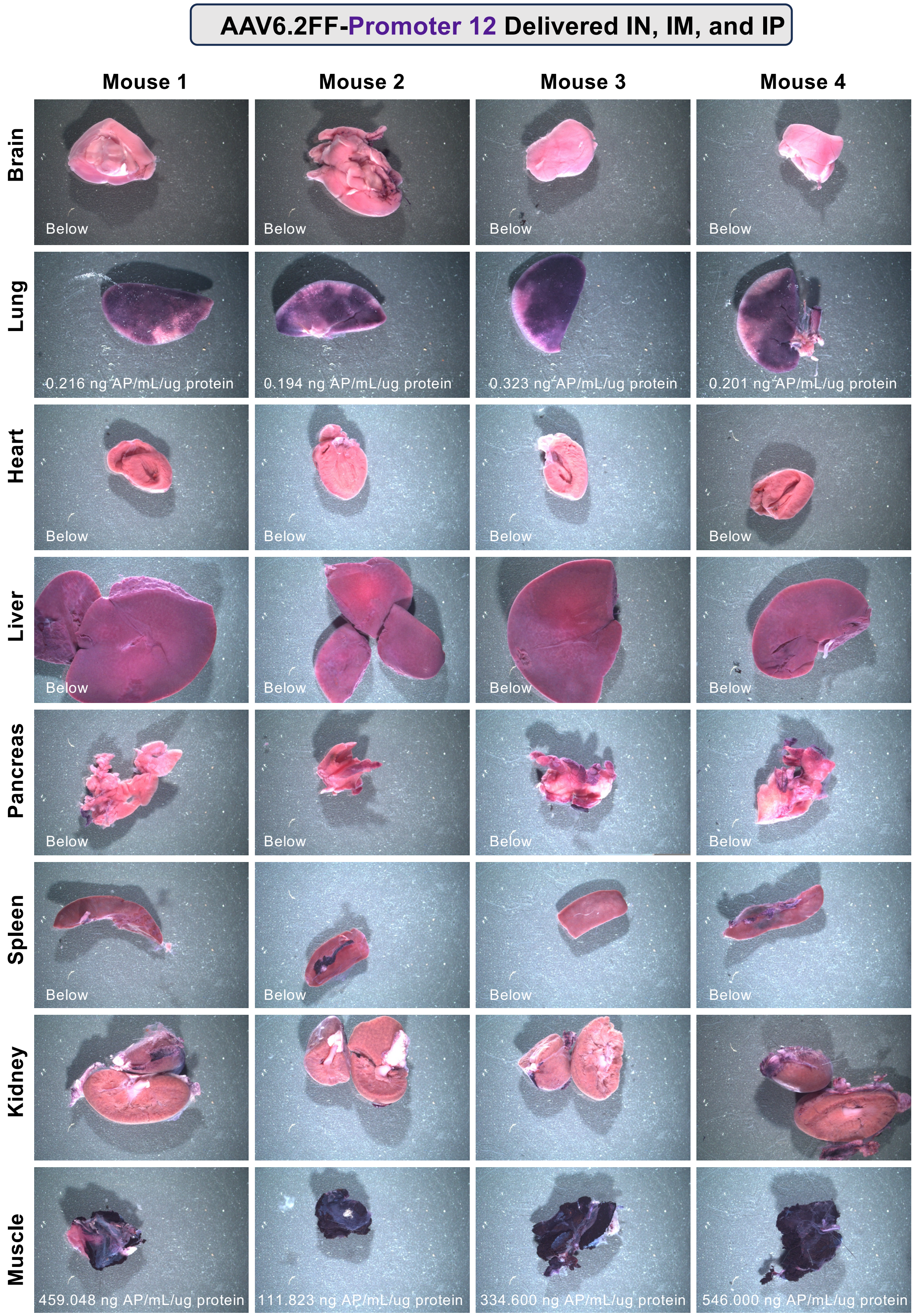

Figure S14. Tissue-wide macroscopic and quantitative analysis of AAV6.2FF-mediated AP expression driven by promoter 12 delivered intranasally, intramuscularly, and intraperitoneally. C57BL/6 mice received 1x10^{11} vg (per route) of AAV6.2FF expressing alkaline phosphatase (AP) driven by promoter 12 via intranasal (IN), intramuscular (IM), and intraperitoneal (IP) administration. At three weeks post-AAV administration, mice were euthanized, and tissues were collected for either macroscopic AP staining or quantitative AP activity analysis. Shown are gross images from all mice and corresponding AP expression levels (ng AP/mL/μg protein) for each tissue.

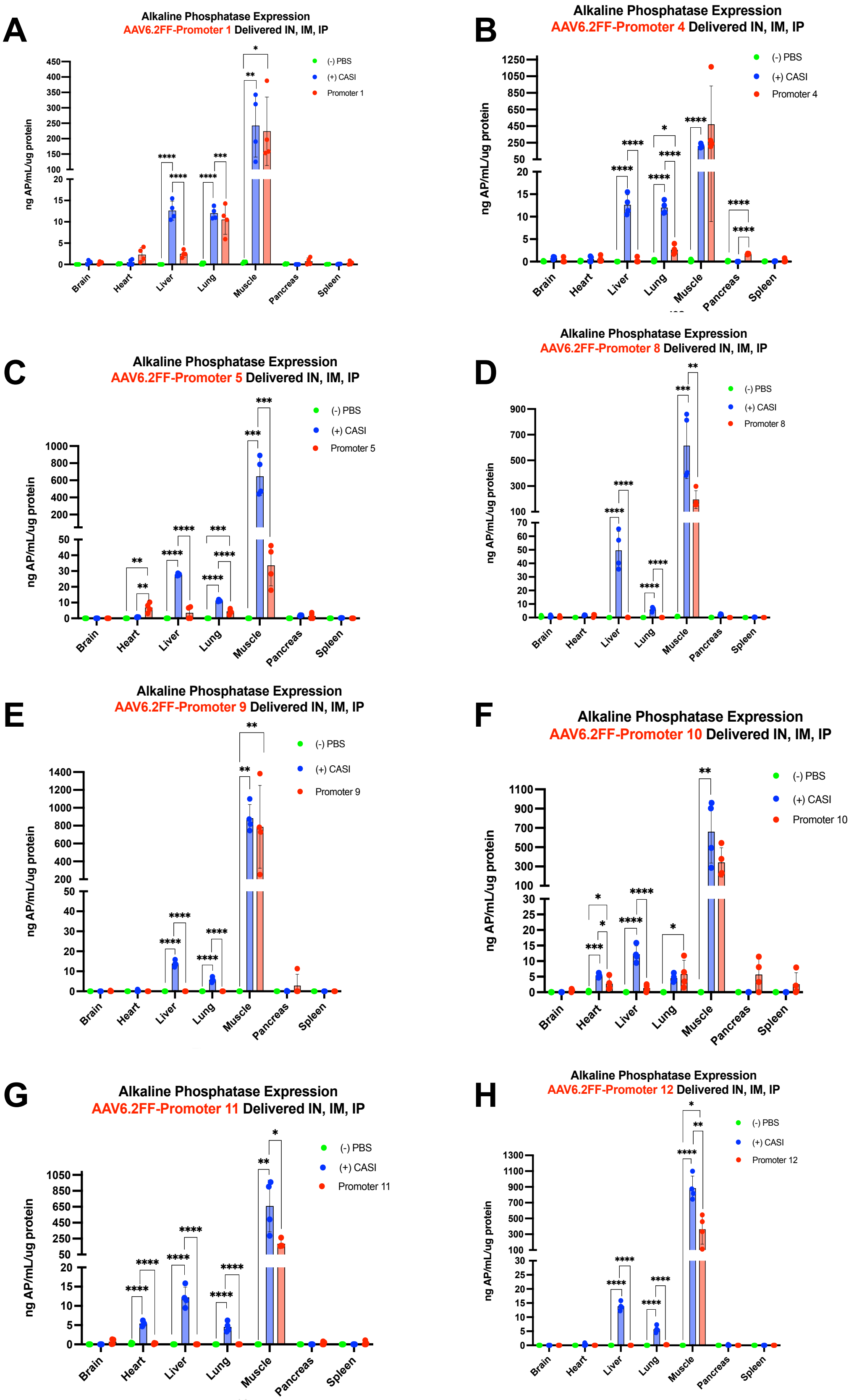

Figure S15. Quantitative analysis of tissue-wide AAV6.2FF-mediated AP expression driven by promoters 1, 4, 5, 8, 9, 10, 11 and 12 delivered intranasally, intramuscularly, and intraperitoneally. Mice received AAV6.2FF-AP driven by promoters (A) 1, (B) 4, (C) 5, (D) 8, (E) 9, (F) 10, (G) 11, or (H) 12, intranasally, intramuscularly and intraperitoneally at a dose of 1×10^{11} vg for each route of administration. Positive and negative control groups received AAV6.2FF-CASI-AP and PBS, respectively. Mice were euthanized 21 days post-AAV administration, tissues were collected, and a portion of each tissue was homogenized. Alkaline phosphatase expression was quantified across tissues using an enzymatic AP activity assay Individual one-way ANOVAs were performed to compare the groups within each tissue. *P ≤ 0.05, ** P ≤ 0.01, *** P ≤ 0.001, and **** P ≤ 0.0001.

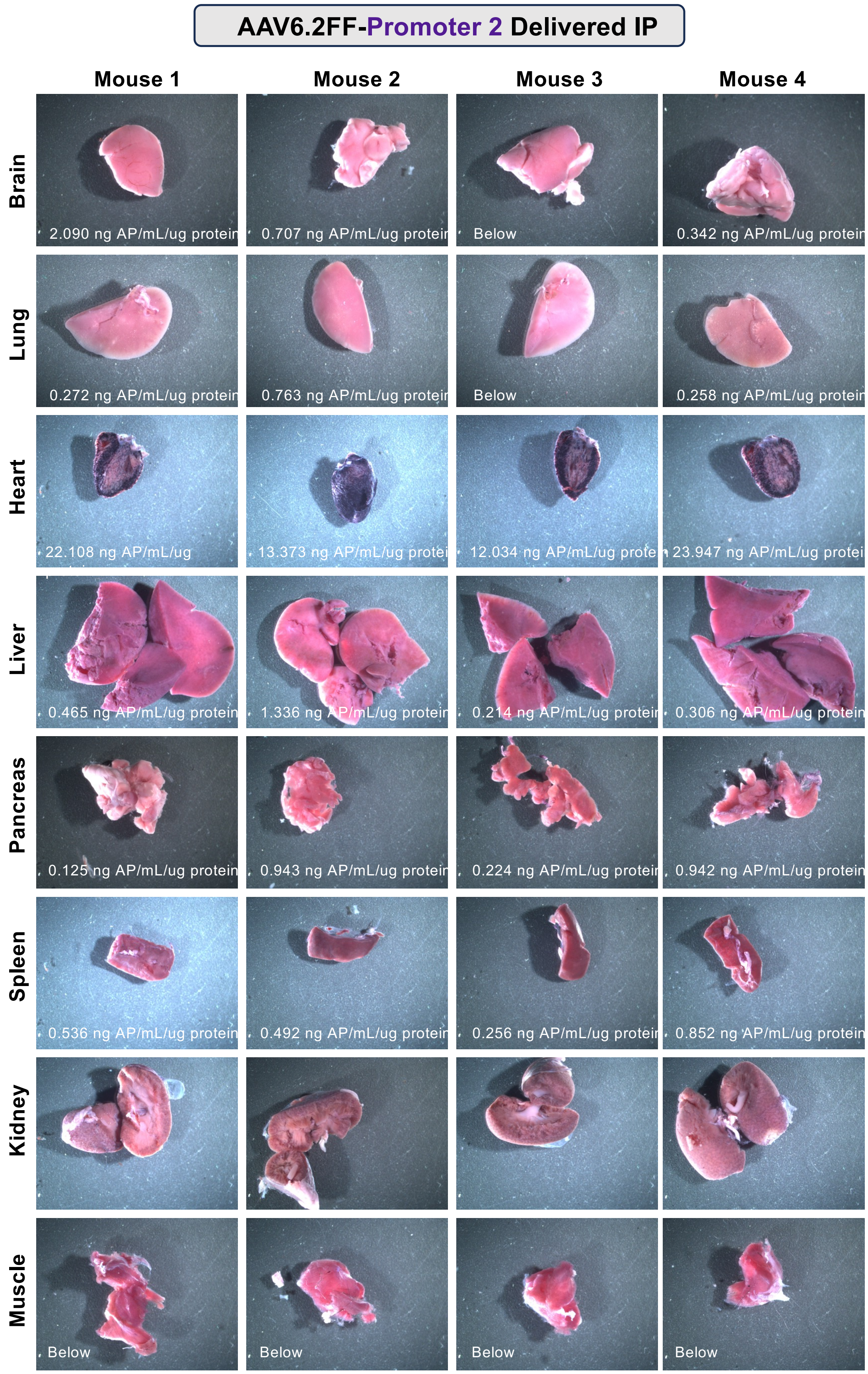

Figure S16. Tissue-wide macroscopic and quantitative analysis of AAV6.2FF-mediated AP expression driven by promoter 2 delivered intraperitoneally. C57BL/6 mice received 1x10^{11} vg of AAV6.2FF expressing alkaline phosphatase (AP) driven by promoter 2 via intraperitoneal (IP) administration only. At three weeks post-AAV administration, mice were euthanized, and tissues were collected for either macroscopic AP staining or quantitative AP activity analysis. Shown are gross images from all mice and corresponding AP expression levels (ng AP/mL/μg protein) for each tissue.

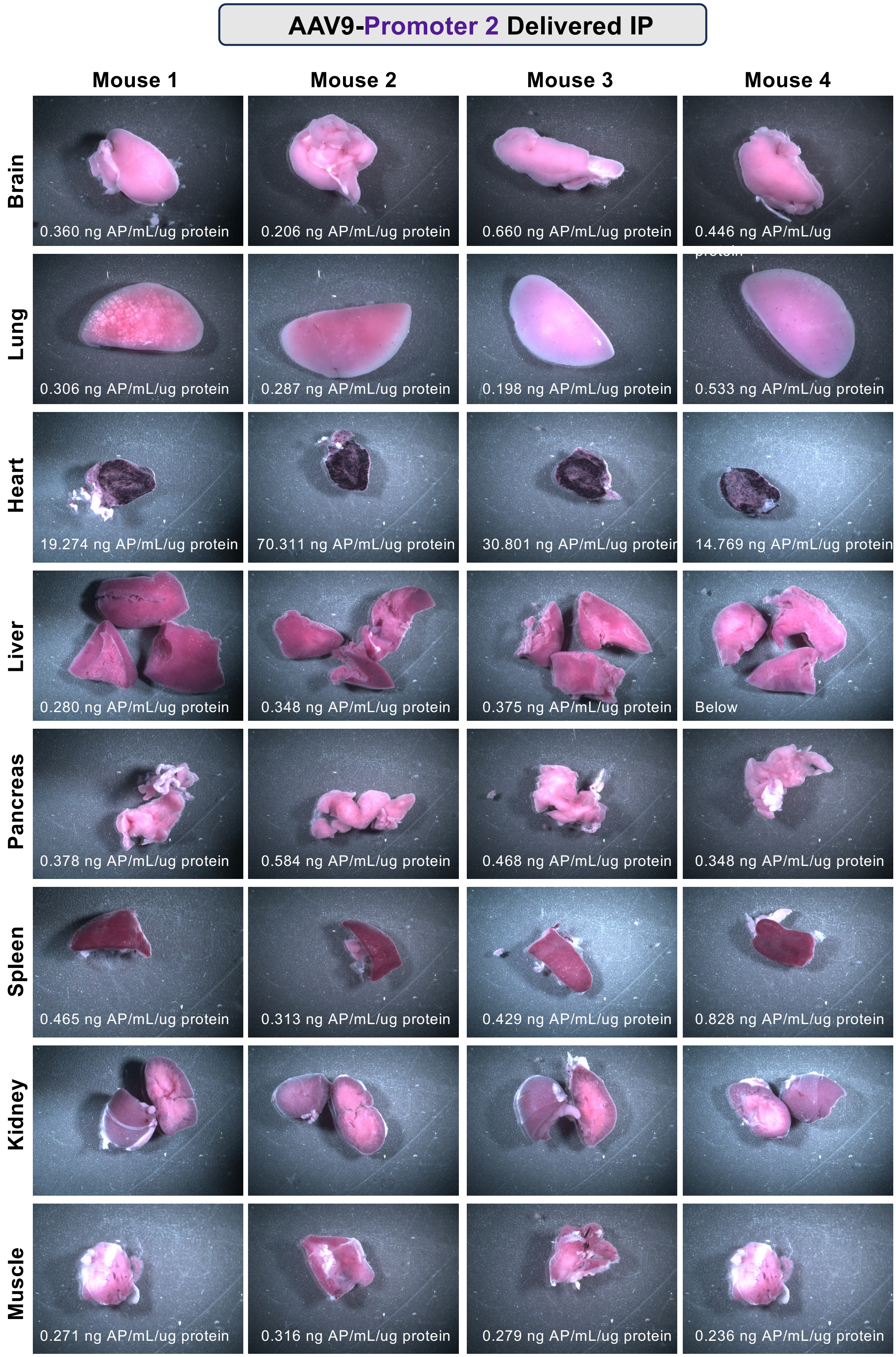

Figure S17. Tissue-wide macroscopic and quantitative analysis of AAV9-mediated AP expression driven by promoter 2 delivered intraperitoneally. C57BL/6 mice received 1x10^{11} vg of AAV9 expressing alkaline phosphatase (AP) driven by promoter 2 via intraperitoneal (IP) administration only. At three weeks post-AAV administration, mice were euthanized, and tissues were collected for either macroscopic AP staining or quantitative AP activity analysis. Shown are gross images from all mice and corresponding AP expression levels (ng AP/mL/μg protein) for each tissue.

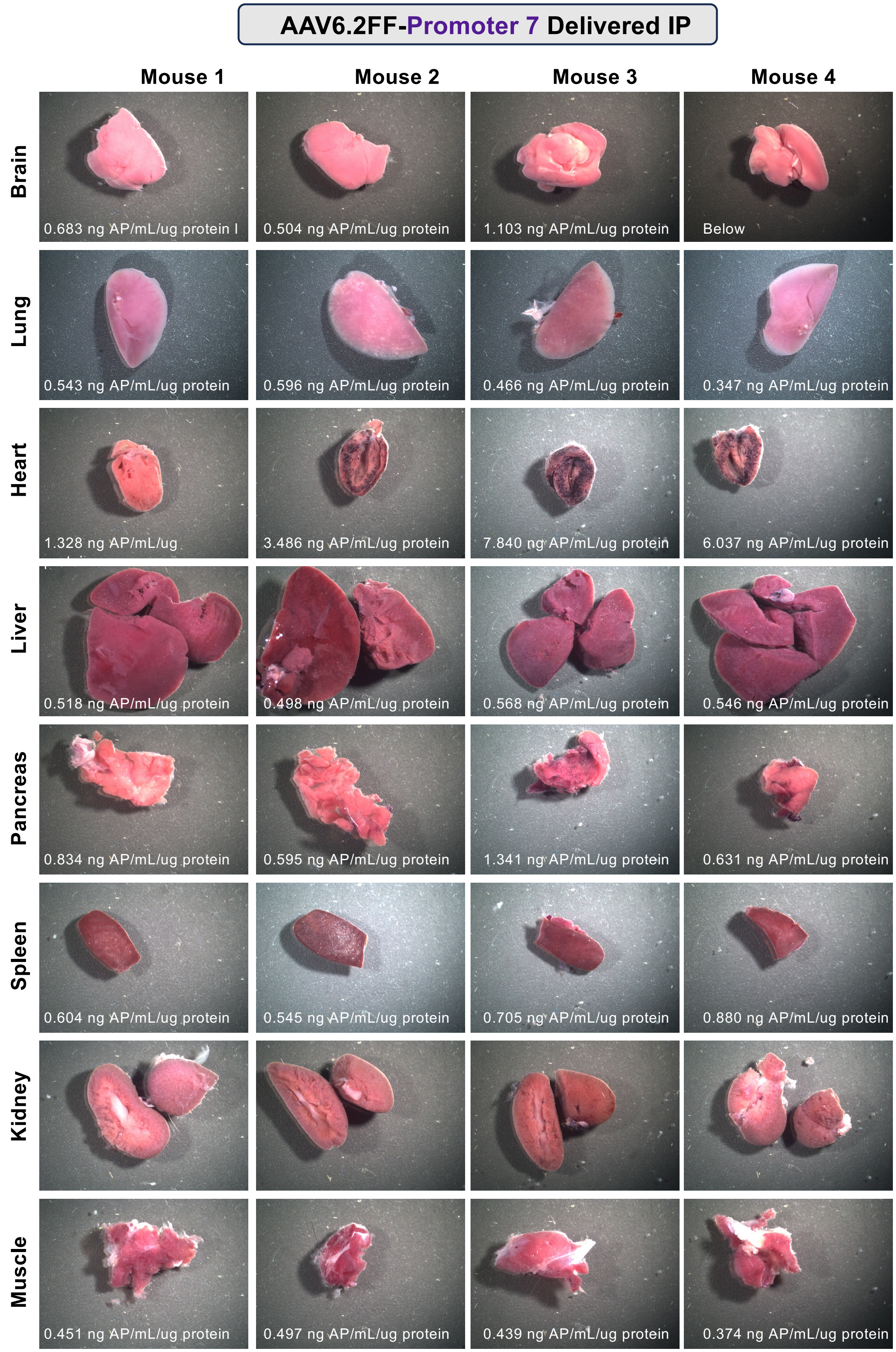

Figure S18. Tissue-wide macroscopic and quantitative analysis of AAV6.2FF-mediated AP expression driven by promoter 7 delivered intraperitoneally. C57BL/6 mice received 1x10^{11} vg of AAV6.2FF expressing alkaline phosphatase (AP) driven by promoter 7 via intraperitoneal (IP) administration only. At three weeks post-AAV administration, mice were euthanized, and tissues were collected for either macroscopic AP staining or quantitative AP activity analysis. Shown are gross images from all mice and corresponding AP expression levels (ng AP/mL/μg protein) for each tissue.

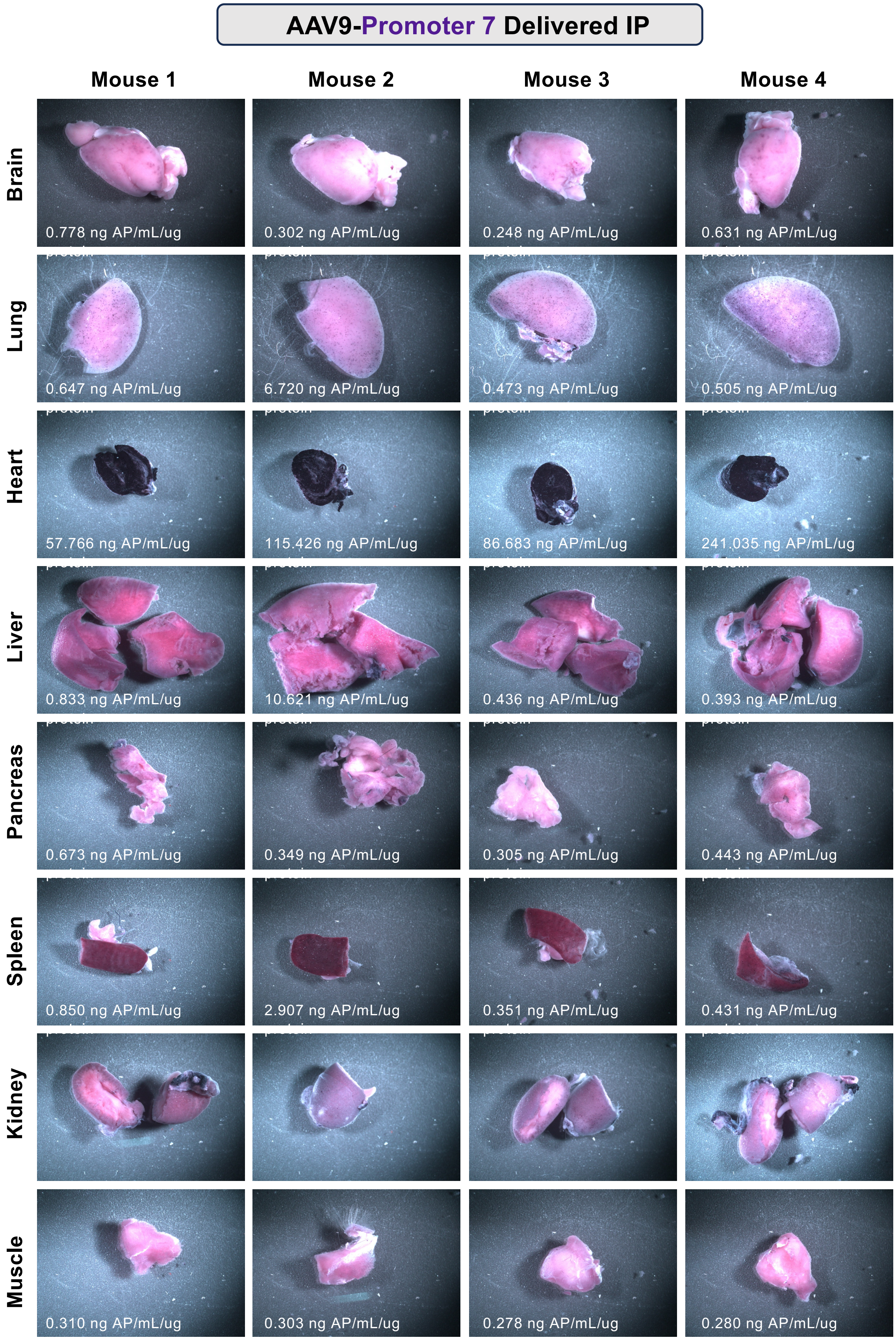

Figure S19. Tissue-wide macroscopic and quantitative analysis of AAV9-mediated AP expression driven by promoter 7 delivered intraperitoneally. C57BL/6 mice received $1x10^{11}$ vg of AAV9 expressing alkaline phosphatase (AP) driven by promoter 7 via intraperitoneal (IP) administration only. At three weeks post-AAV administration, mice were euthanized, and tissues were collected for either macroscopic AP staining or quantitative AP activity analysis. Shown are gross images from all mice and corresponding AP expression levels (ng AP/mL/µg protein) for each tissue.

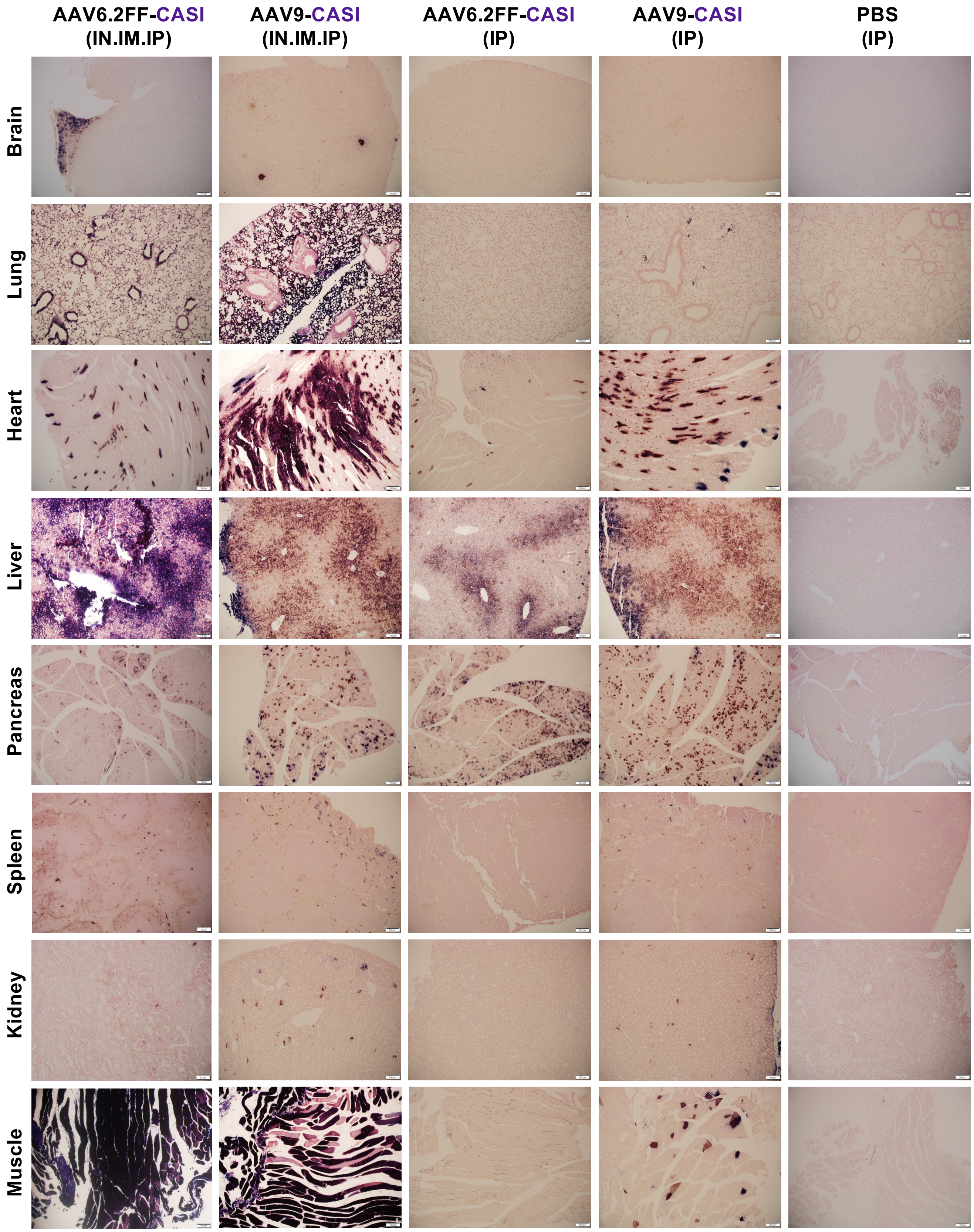

Figure S20. Histological analysis of AAV-mediated AP expression driven by the CASI promoter across different AAV serotypes and different routes of administration. Mice received either AAV6.2FF-AP or AAV9-AP driven by the CASI promoter administered via either combined routes [intranasal (IN), intramuscular (IM), and intraperitoneal (IP)] or a single intraperitoneal injection at a dose of 1×10^{11} vg per route. Mice were euthanized 21 days post AAV administration, and tissues were collected, fixed and stained for AP. Tissues were subsequently embedded in paraffin, sectioned and stained to detect AP expression, with nuclei counterstained using nuclear fast red. Representative images taken at 10x magnification are shown.

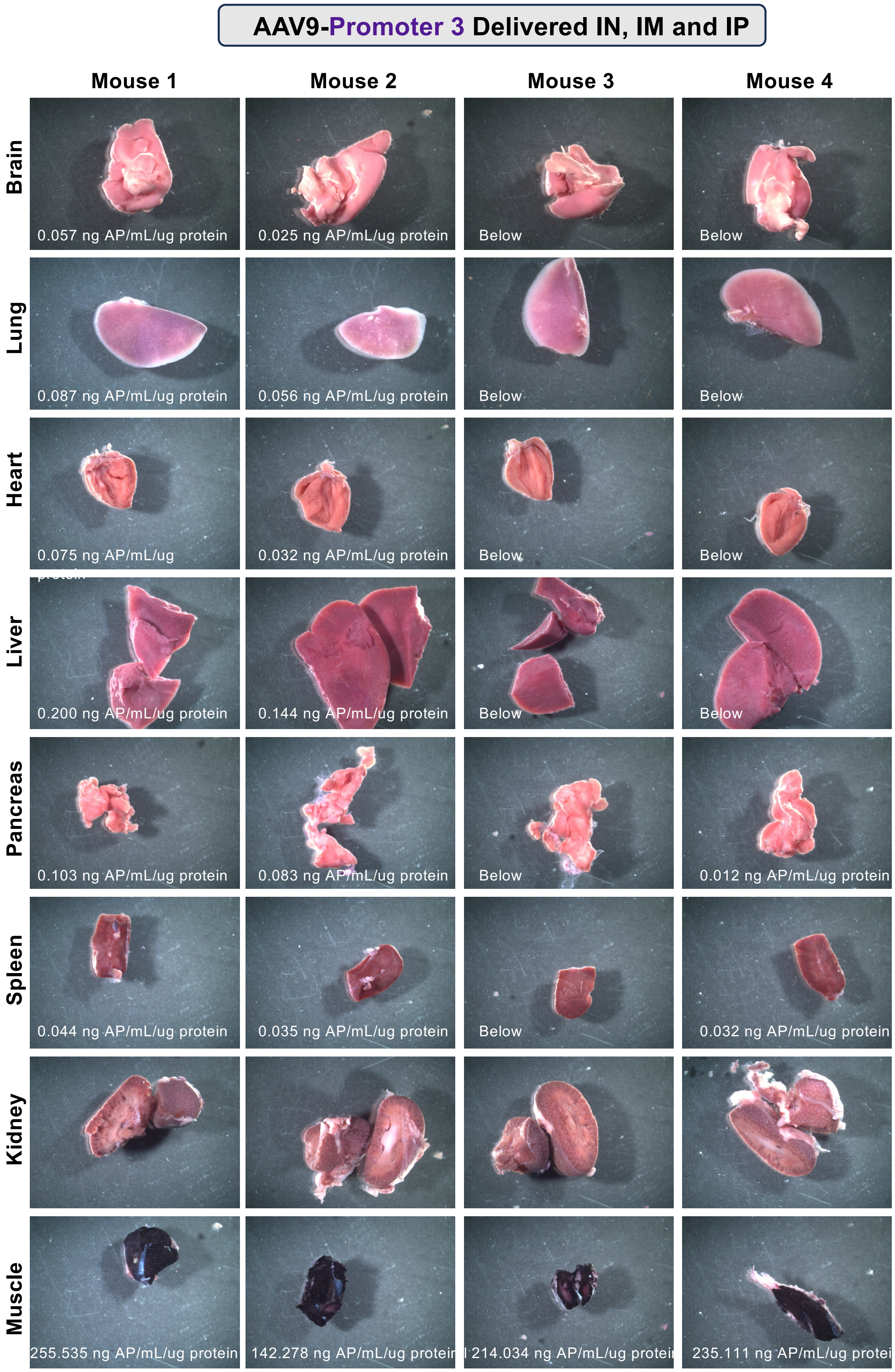

Figure S21. Tissue-wide macroscopic and quantitative analysis of AAV9-mediated AP expression driven by promoter 3 delivered intranasally, intramuscularly, and intraperitoneally. C57BL/6 mice received AAV9 expressing alkaline phosphatase (AP) driven by promoter 3 via intranasal (IN), intramuscular (IM) and intraperitoneal (IP) administration at a dose of $1x10^{11}$ vg per route. Three weeks post-AAV, mice were euthanized, and tissues were collected for either macroscopic AP staining or quantitative AP activity analysis. Shown are gross images from all mice and corresponding AP expression levels (ng AP/mL/μg protein) for each tissue.

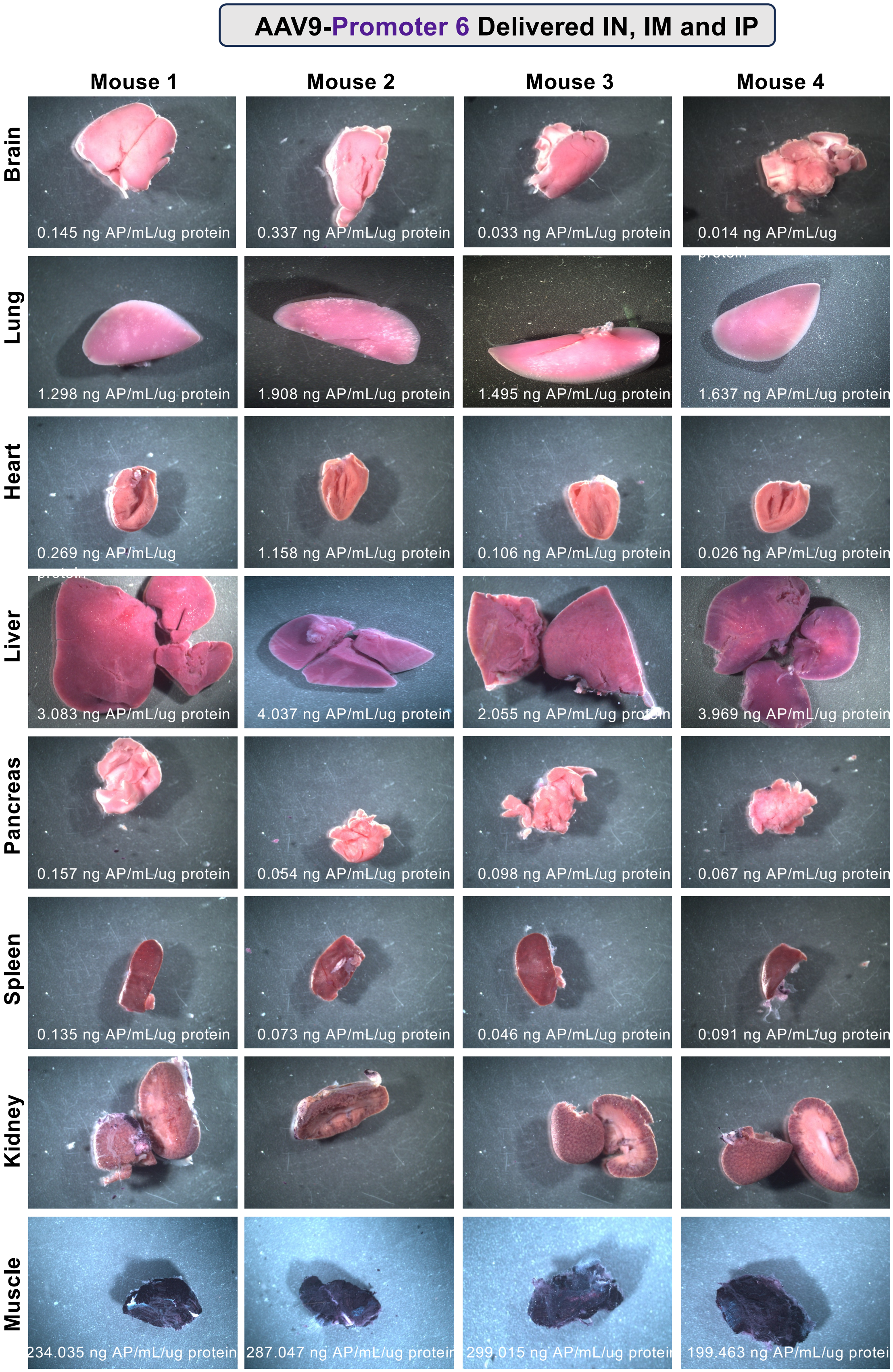

Figure S22. Tissue-wide macroscopic and quantitative analysis of AAV9-mediated AP expression driven by promoter 6 delivered intranasally, intramuscularly, and intraperitoneally. C57BL/6 mice received AAV9 expressing alkaline phosphatase (AP) driven by promoter 6 via intranasal (IN), intramuscular (IM) and intraperitoneal (IP) administration at a dose of $1x10^{11}$ vg per route. Three weeks post-AAV, mice were euthanized, and tissues were collected for either macroscopic AP staining or quantitative AP activity analysis. Shown are gross images from all mice and corresponding AP expression levels (ng AP/mL/μg protein) for each tissue.

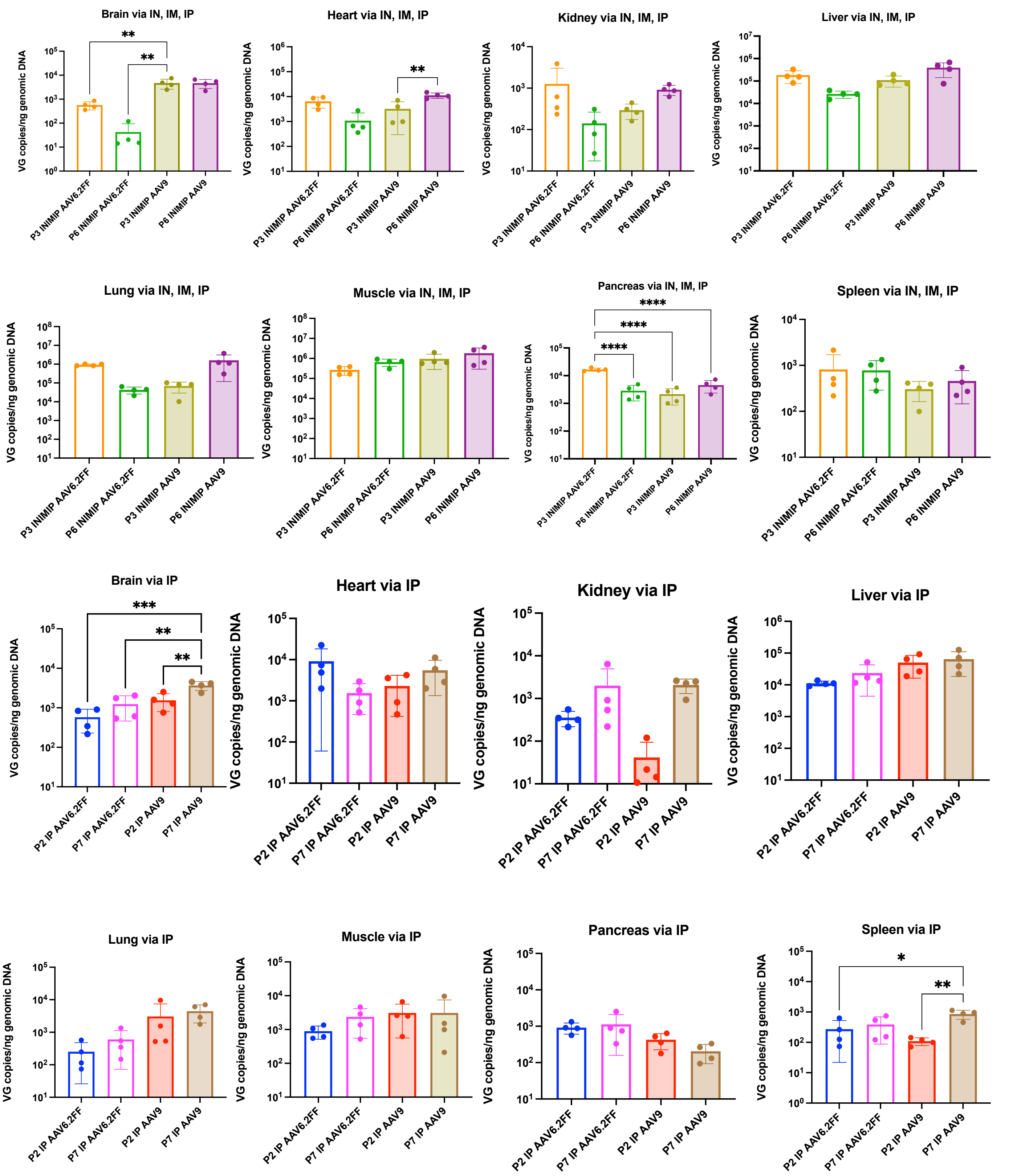

Figure S23. Tissue-wide AAV vector genome quantification. DNA was extracted from paraffin-embedded tissue sections, and vector genome copies were quantified by qPCR, normalized to total genomic DNA (vg/ng) and corrected by subtracting background levels measured in each negative control tissue sample. The shaded bars represent mice that received the AAV9 capsid expressing alkaline phosphatase (AP) from promoters 2, 3, 6, or 7 by either combined intranasal (IN), intramuscular (IM), and intraperitoneal (IP) administration or IP administration only, at a dose of 1×10^{11} vg per route. Unshaded bars represent mice that received AAV6.2FF expressing AP from promoters 2, 3, 6 or 7 by either combined IN, IM, IP administration or IP administration only, at a dose of 1×10^{11} vg per route. Individual one-way ANOVAs were performed to compare the groups within each tissue. *$P \leq 0.05$, ** $P \leq 0.01$, *** $P \leq 0.001$, and **** $P \leq 0.0001$..
